# Investigating Genetic and Other Determinants of First-Onset Myocardial Infarction in Malaysia: Protocol for the Malaysian Acute Vascular Events Risk Study

**DOI:** 10.2196/31885

**Published:** 2022-02-10

**Authors:** Rajiv Chowdhury, Mohd Fairulnizal Md Noh, Sophia Rasheeqa Ismail, Kim Robin van Daalen, Puteri Sofia Nadira Megat Kamaruddin, Siti Hafizah Zulkiply, Nur Hayati Azizul, Norhayati Mustafa Khalid, Azizan Ali, Izyan Mohd Idris, Yong Shih Mei, Shazana Rifham Abdullah, Norfashihah Faridus, Nur Azirah Md Yusof, Nur Najwa Farahin M Yusoff, Rahman Jamal, Aizai Azan Abdul Rahim, Abdul Kahar Abdul Ghapar, Ammu Kutty Radhakrishnan, Alan Yean Yip Fong, Omar Ismail, Saravanan Krishinan, Chuey Yan Lee, Liew Houng Bang, Eashwary Mageswaren, Kauthaman Mahendran, Nor Hanim Mohd Amin, Gunavathy Muthusamy, Aaron Ong Hean Jin, Ahmad Wazi Ramli, Noel Thomas Ross, Anwar Irawan Ruhani, Mansor Yahya, Yusniza Yusoff, Siti Khairani Zainal Abidin, Laryssa Amado, Thomas Bolton, Sophie Weston, Jason Crawte, Niko Ovenden, Ank Michielsen, Md Mostafa Monower, Wan Rozita Wan Mahiyuddin, Angela Wood, Emanuele Di Angelantonio, Nur Suffia Sulaiman, John Danesh, Adam S Butterworth

**Affiliations:** 1 British Heart Foundation Cardiovascular Epidemiology Unit Department of Public Health and Primary Care University of Cambridge Cambridge United Kingdom; 2 Centre for Non-Communicable Disease Research Dhaka Bangladesh; 3 Institute for Medical Research, National Institute of Health, Ministry of Health Kuala Lumpur Malaysia; 4 UKM Medical Molecular Biological Institute Kuala Lumpur Malaysia; 5 National Heart Institute Kuala Lumpur Malaysia; 6 Department of Cardiology Hospital Serdang Selangor Malaysia; 7 Jeffery Cheah School of Medicine and Health Sciences Monash University Malaysia Subang Jaya Malaysia; 8 Department of Cardiology, Sarawak Heart Centre Kota Samarahan Malaysia; 9 Clinical Research Centre, Institute for Clinical Research, Sarawak General Hospital Kuching Malaysia; 10 Department of Cardiology Hospital Pulau Pinang Pulau Pinang Malaysia; 11 Department of Cardiology Hospital Sultanah Bahiyah Kedah Malaysia; 12 Department of Cardiology Hospital Sultanah Aminah Johor Malaysia; 13 Department of Cardiology & Clinical Research Centre Hospital Queen Elizabeth II Sabah Malaysia; 14 Department of General Medicine Hospital Tengku Ampuan Rahimah Selangor Malaysia; 15 Department of General Medicine & Clinical Research Centre Hospital Melaka Melaka Malaysia; 16 Department of General Medicine Hospital Raja Permaisuri Bainun Perak Malaysia; 17 Department of General Medicine Hospital Shah Alam Selangor Malaysia; 18 Department of General Medicine Hospital Tuanku Fauziah Perlis Malaysia; 19 Department of Cardiology Hospital Sultanah Nur Zahirah Terengganu Malaysia; 20 Department of General Medicine Hospital Kuala Lumpur Kuala Lumpur Malaysia; 21 Department of Cardiology Hospital Tengku Ampuan Afzan Pahang Malaysia; 22 Department of General Medicine Hospital Sungai Buloh Selangor Malaysia; 23 National Institute for Health Research Blood and Transplant Research Unit in Donor Health and Genomics, University of Cambridge Cambridge United Kingdom; 24 National Heart Foundation Hospital & Research Institute, Mirpur Dhaka Bangladesh; 25 Health Data Research UK Cambridge, Wellcome Genome Campus and University of Cambridge Cambridge United Kingdom; 26 British Heart Foundation Centre of Research Excellence, University of Cambridge Cambridge United Kingdom; 27 Department of Human Genetics, Wellcome Sanger Institute Hinxton United Kingdom

**Keywords:** myocardial infarction, cardiovascular disease, case-control study, Malaysia

## Abstract

**Background:**

Although the burden of premature myocardial infarction (MI) is high in Malaysia, direct evidence on the determinants of MI in this multi-ethnic population remains sparse.

**Objective:**

The Malaysian Acute Vascular Events Risk (MAVERIK) study is a retrospective case-control study established to investigate the genomic, lipid-related, and other determinants of acute MI in Malaysia. In this paper, we report the study protocol and early results.

**Methods:**

By June 2019, we had enrolled approximately 2500 patients with their first MI and 2500 controls without cardiovascular disease, who were frequency-matched by age, sex, and ethnicity, from 17 hospitals in Malaysia. For each participant, serum and whole blood have been collected and stored. Clinical, demographic, and behavioral information has been obtained using a 200-item questionnaire.

**Results:**

Tobacco consumption, a history of diabetes, hypertension, markers of visceral adiposity, indicators of lower socioeconomic status, and a family history of coronary disease were more prevalent in cases than in controls. Adjusted (age and sex) logistic regression models for traditional risk factors indicated that current smoking (odds ratio [OR] 4.11, 95% CI 3.56-4.75; *P*<.001), previous smoking (OR 1.34, 95% CI 1.12-1.60; *P*=.001), a history of high blood pressure (OR 2.13, 95% CI 1.86-2.44; *P*<.001), a history of diabetes mellitus (OR 2.72, 95% CI 2.34-3.17; *P*<.001), a family history of coronary heart disease (OR 1.28, 95% CI 1.07-1.55; *P*=.009), and obesity (BMI >30 kg/m^2^; OR 1.19, 95% CI 1.05-1.34; *P*=.009) were associated with MI in age- and sex-adjusted models.

**Conclusions:**

The MAVERIK study can serve as a useful platform to investigate genetic and other risk factors for MI in an understudied Southeast Asian population. It should help to hasten the discovery of disease-causing pathways and inform regionally appropriate strategies that optimize public health action.

**International Registered Report Identifier (IRRID):**

RR1-10.2196/31885

## Introduction

Coronary heart disease (CHD), with myocardial infarction (MI) as a key clinical manifestation, is the leading cause of death in Malaysia and elsewhere in Southeast Asia [1,2]. In recent decades, Malaysia has experienced a sustained increase in the incidence of CHD [3]. According to World Health Organization estimates, the age-adjusted death rates of CHD are approximately twice as high in Malaysia compared with the United States or the United Kingdom [4]. Furthermore, studies in Malaysia and other Southeast Asian countries indicate that CHD events tend to occur at younger ages and are characterized by more severe clinical features than in Western populations [5,6], resulting in substantial losses in productive working years owing to death and disability. Indeed, CHD in Malaysia is characterized by high rates of short-term post-MI mortality [7]. However, there is limited direct evidence regarding the determinants of CHD in Malaysia [2]. Therefore, we have established the Malaysian Acute Vascular Events Risk (MAVERIK) retrospective case-control study of MI to investigate genomic, lipid-related, and other determinants of acute MI in Malaysia. In this paper, we describe the rationale, protocol, and early baseline results of this study.

## Methods

**Figure 1:**
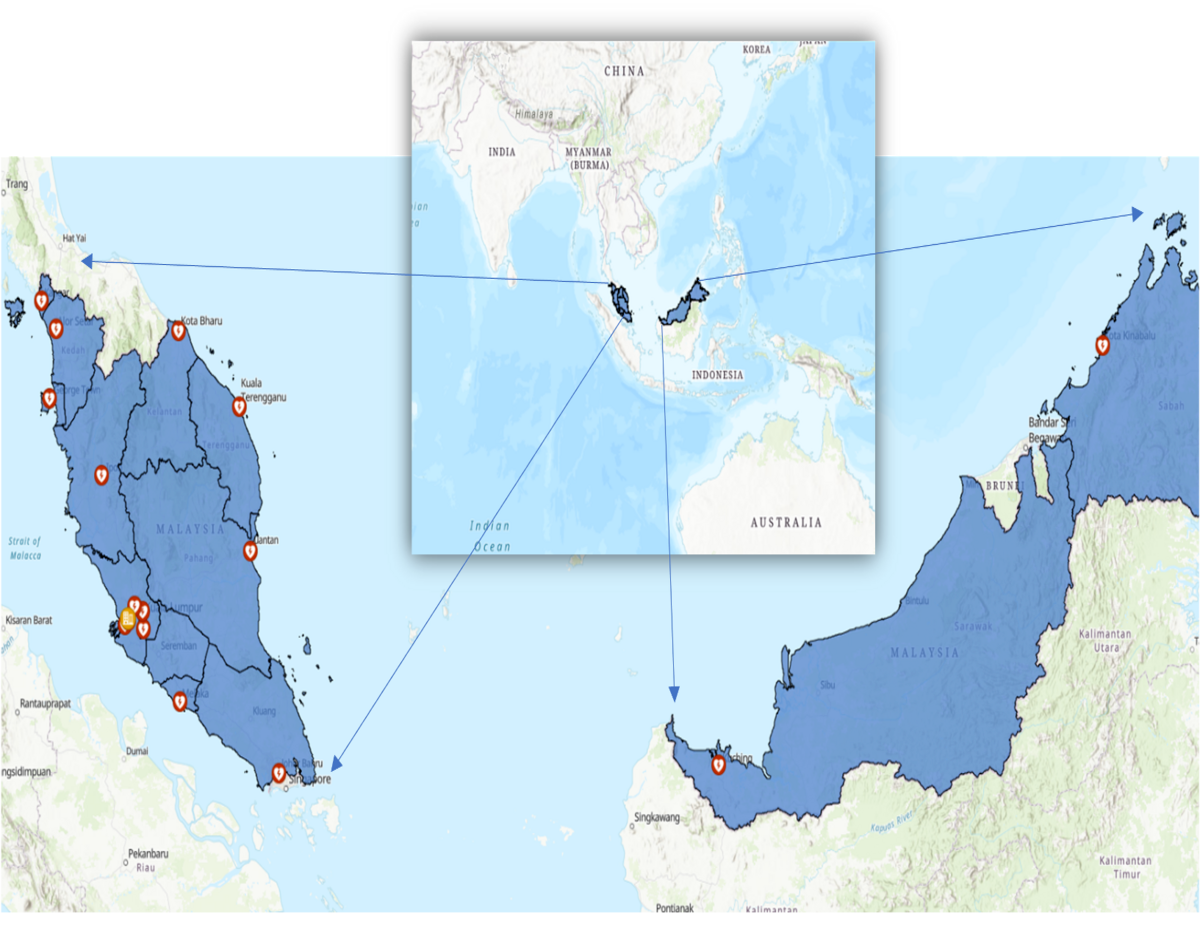
Location of the Malaysian Acute Vascular Events Risk (MAVERIK) study hospital sites. The sites in red denote the recruitment centers: Hospital Tuanku Fauziah Kangar, Hospital Sultanah Bahiyah Alor Setar, Hospital Pulau Pinang, Hospital Raja Permaisuri Bainun Ipoh, Hospital Serdang, Hospital Kuala Lumpur, Hospital Shah Alam, Hospital Tengku Ampuan Rahimah Klang, Hospital Sungai Buloh, Hospital Melaka, Hospital Sultanah Aminah Johor Bharu, Hospital Tengku Ampuan Afzan Kuantan, Hospital Sultanah Nur Zahirah Kuala Terengganu, Hospital Raja Perempuan Zainab II Kota Bharu, Hospital Queen Elizabeth II Kota Kinabalu, Hospital Umum Sarawak, and Pusat Jantung Sarawak Kota Samarahan. Sites in yellow denote the local coordinating center and national sample repository of the Institute for Medical Research, Malaysia. This figure has been made using Esri ArcGIS Online with the Topographic Basemap.

The MAVERIK study was established in 2017 in collaboration with the British Heart Foundation Cardiovascular Epidemiology Unit at the University of Cambridge, United Kingdom (the study’s international coordinating center); the Institute for Medical Research (IMR), Malaysia (the study’s National Collaborating center); and 17 national collaborating cardiology referral hospitals in Malaysia serving as the study’s recruitment centers (encompassing nearly all states and federal territories in Malaysia; Figure 1).

### Study Design and Participants

**Figure 2:**
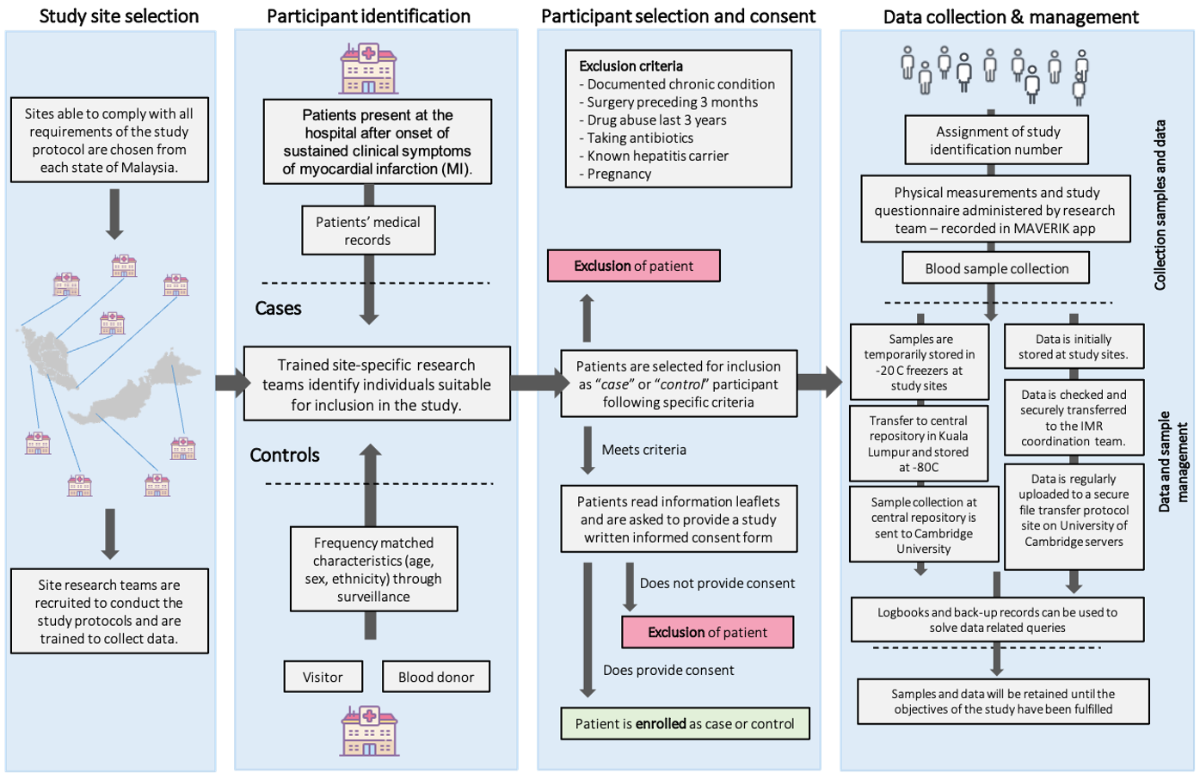
The Malaysian Acute Vascular Events Risk (MAVERIK) Study flow diagram for activities.

The MAVERIK study is a retrospective case-control study of acute MI (Figure 2). By August 2019, the study had recruited 2547 confirmed first-ever MI cases and 2500 healthy controls without cardiovascular disease (CVD). Patients admitted to the cardiology unit or wards, general medical wards, or coronary care units of the participating hospitals have been screened by study research assistants trained to identify all relevant cases of acute MI. Cases were eligible for inclusion in the study if they (1) were Malaysians residing in Malaysia; (2) were adults aged ≥18 years; (3) were present at the hospital after the onset of clinical symptoms suggestive of MI, with symptoms lasting longer than 20 minutes; (4) had a confirmed diagnosis of acute MI based on the Malaysian Clinical Practise Guidelines [8-10], which included levels of cardiac troponins above the 99th percentile of the upper reference limits, accompanied with at least one of the following: clinical history consistent with chest pain of ischemic origin, new ischemic electrocardiogram changes or development of pathological Q waves, imaging evidence of new loss of viable myocardium or new regional wall motion abnormality, or identification of an intracoronary thrombus by angiography; (5) had no previous CVD, defined as self-reported history of MI, coronary revascularization, transient ischemic attack, stroke, other CVDs or evidence of CHD on a prior electrocardiogram, or in other medical records; and (6) were not concurrently hospitalized for any MI or CVD events.

Control participants were recruited within 3 months of recruiting the index cases and were frequency-matched to cases by age (in 5-year age bands), sex, and self-reported ethnicity (Malay, Chinese, Indian, Sabah and Sarawak Bumiputera, Orang Asli, and others). The control participants had no previous self-reported history of CVD and were identified in the same hospitals as the index cases or collaborating hospitals in the same region and were recruited in the following order of priority: (1) visitors who were accompanying patients attending the outpatient department; (2) visitors of inpatients who were not part of the MAVERIK study; (3) visitors of index MI cases who were not their blood relatives; and (4) healthy blood donors in the same hospital. The selected cases and controls were excluded if they met any of the following criteria: (1) had documented chronic conditions on past medical history, such as malignancy, kidney (estimated glomerular filtration rate [eGFR] of <60 mL/min/1.73 m^2^), thyroid or inflammatory disorders, or any chronic infection on past medical history; (2) had a recent history of surgery in the last 3 months; (3) were taking antibiotics; (4) were known to abuse drugs in the last 3 years; (5) were pregnant; or (6) were unwilling or unable to provide consent.

Before adopting the approach for the selection of controls, we carefully assessed several other options, such as choosing control groups who had an unrelated disease, population-based community controls, and controls from occupational settings or health-check clinics. Our chosen approach was considered desirable because it achieved a balance between feasibility and scientific rigor and because it was scalable in Malaysia. By contrast, even though the use of population-based community controls may be desirable in principle, it is considerably more labor-intensive and expensive. Furthermore, in agreement with the Wellcome Trust Case-Control Consortium [11], the MAVERIK study controls can be efficiently and validly used, at least in genetic studies, for patients with other cardiometabolic conditions owing to the broad geographic and ethnic scope of the MAVERIK study.

### Questionnaire Administration and Physical Measurements

The MAVERIK study questionnaires used are adapted to the Malaysian context based on questionnaires used in previous case-control studies of acute MI in Southeast Asian populations (eg, the Bangladesh Risk of Acute Vascular Events study in Bangladesh) and were piloted in Malaysia before implementation in the MAVERIK study [12,13]. Trained personnel administered a prepiloted epidemiological questionnaire seeking information on >200 items related to demographic characteristics, consanguinity, behavior (eg, tobacco and alcohol consumption, dietary intake, and physical activity), personal and family medical history, and medication use (Table 1). A copy of the study questionnaire is available in the Multimedia Appendix 1. Paper-free data collection involved a bespoke Android interface (*MAVERIK app*) and operated through handheld touchscreen tablet devices. For a subset of the cases and controls, data were collected using both electronic and paper questionnaires for quality comparison purposes. To assess local dietary patterns, an 83-item food-frequency questionnaire, with an estimated standard portion size assigned to each food item, was adapted from a validated Malaysian national survey questionnaire (Multimedia Appendix 1, Table S1) [14]. We used standardized procedures and equipment to assess height, weight, waist and hip circumference, systolic and diastolic blood pressure, and heart rate. Waist circumference was assessed over the abdomen at the widest diameter between the costal margin and the iliac crest, and hip circumference at the level of the greater trochanters (ie, the widest diameter around the buttocks). For both index cases and controls, anthropometric measurements were performed in a standing position (a copy of the anthropometric measurement protocol is available in the Multimedia Appendix 1).

**Table 1:** Summary of questionnaire-based information, physical measurements, and coronary assessments collected in the Malaysian Acute Vascular Events Risk study.

Characteristics	Availability of information
Demographic and behavioral	Age at onset, gender, use of tobacco for smoking or chewing, use of tobacco alternatives (vaping and e-cigarettes), levels of physical activity (sedentary, moderate, and vigorous), detailed dietary habits
Sociodemographic	Education, occupation, income (participant and household), marital status, consanguinity (of parents or with own spouse), residence (urban and rural)
Signs and symptoms	Time thrombolysis initiated, type of MI^a^, outcome for the current event, onset of symptoms, hospital arrival, health care contact
Personal and family medical history (self-reported)	High blood pressure, high blood cholesterol, diabetes mellitus and family history of CHD^b^, stroke, and sudden cardiac death
Sleep patterns and cell phone use	Hours of sleep, napping, time spent receiving or making phone calls and other purposes
Physical measurements	Blood pressure (systolic and diastolic), heart rate, height, weight, waist, and hip circumferences

^a^MI: myocardial infarction.

^b^CHD: coronary heart disease.

### General Quality Control Approaches

To ensure effective surveillance for eligible MI cases and controls, the research staff received training and supervision by a study medical coordinator designated for that center. We aimed to reduce variation in the collection of data through extensive training, the use of standardized approaches, validated instruments, and paper-free methods with built-in validity checks and queries. For example, our paper-free data collection tool involved extensive computerized checks to restrict missing values, duplications, inconsistencies, and outliers. Information from the study app was transferred daily in a secure manner to the central database at the IMR, with a copy also kept at the UK coordinating center for additional checks and queries.

### Collection and Storage of Biological Samples

A total of 20 mL of nonfasting whole blood was collected from each participant (with the time of their last meal recorded) in 2×5 mL designated serum tubes and 2×5 mL designated EDTA tubes. For MI cases, we recorded the time passed since the onset of pain and the administration of thrombolytic medication. Samples are centrifuged (2500 g for 15 minutes) within 45 minutes of venipuncture. Isolated serum, EDTA plasma, and whole blood samples were stored in cryogenic vials at the local recruiting centers in −80 °C freezers or temporarily in −20 °C freezers until transfer to −80 °C freezers (Multimedia Appendix 1, Figure S1). Samples have been transferred to the central repository at the IMR in Kuala Lumpur, typically within 2 weeks. To enable the measurement of additional potential risk factors (eg, metal contaminants), since October 2018, we have also collected finger- and toe-nail clippings, kept separately in plastic bottles, stored at room temperature, and transferred regularly to the IMR to be weighed and cataloged. Biological samples are stored long-term at both the IMR and the UK coordinating center.

### Ethical Approval and Informed Consent

The MAVERIK study has received approval from the Malaysian Medical Research and Ethics Committee (reference (11)KKM/NIHSEC/P17-103). Written informed consent has been obtained from each participant before recruitment, including for future use of data and stored samples for genetic, biochemical, recall-by-genotype or -phenotype, and other analyses. The data collected in this research are subject to the core data protection principles and requirements of the United Kingdom Data Protection Act 1998 [15]. The investigators and institutional review boards are committed to ensure that research is conducted according to the latest version of the Declaration of Helsinki [16], Universal Declaration on the Human Genome and Human Rights adopted by the United Nations Educational, Scientific and Cultural Organization (UNESCO) [17], and other legislations.

### Statistical Analysis

Sample size considerations were guided by a combination of pragmatic constraints (eg, the availability of resources) and statistical power calculations. For the association with early-onset MI, the study of approximately 2500 cases and 2500 controls should provide 80% power to detect an odds ratio (OR) of 1.08 per 1 SD increase at a 5% significance level. This report includes an initial descriptive analysis in accordance with the Strengthening the Reporting of Observational Studies in Epidemiology guidelines for case-control studies (Multimedia Appendix 1, Table S4) [18].

Continuous variables were summarized using mean (SD) or median and IQR, and categorical variables were summarized using frequencies. Extreme or implausible values for height, weight, waist circumference, hip circumference, BMI, and waist-to-hip ratio were excluded (eg, height<110 cm or >200 cm; weight<25 kg and >450 kg). Variables were compared using the *t* test or Mann-Whitney *U* test for continuous variables and the chi-square test or Fisher exact (n≤5 in any cell) test for categorical variables. All statistical tests were 2-sided, and the significance level was set at *P*<.05. To evaluate the association between MI and selected risk factors, crude and adjusted (sex and age) OR with 95% CIs were calculated using unconditional logistic regression, in keeping with our study’s frequency-matched controls rather than individually matched controls. Analyses were performed using STATA (version 16, StataCorp) and R (version 4.0.1, R Foundation for Statistical Computing). Future statistical analyses will be developed following relevant guidelines for case-control data (eg, Strengthening the Reporting of Observational Studies [18] and National Cancer Institute/National Human Genome Research Institute (NCI/NHGR) working group [19]) and will be presented elsewhere.

## Results

Complete information on age, sex, and ethnicity was available for 2547 MI cases and 2500 controls (Table 2). Missingness for other variables ranged between 2.2% (history of high blood pressure in controls) and 11.6% (monthly income level in cases). Complete case analysis was used (ie, participants with missing values were excluded from the analysis). The mean (SD) age of MI cases was 50.3 (9.4) years, with 91.2% (2324/2547) of participants being male. In total, 60.7% (1548/2547) of cases self-identified as being of Malay ancestry, 19.1% (487/2547) were of Indian ancestry, 13.5% (346/2547) were of Chinese ancestry, and 5.8% (150/2547) as Sabah and Sarawak Bumiputera (including Iban, Kadazan, Dusun, Bidayuh, Melanau, Bumiputera of Sabah, and other Bumiputera of Sarawak ethnicities). As expected, the age, sex, and ethnicity of the cases and controls were similar owing to the frequency matching (standardized mean difference [SMD] age=0.1443 years; SMD female sex=0.0008; SMD Malay ethnicity=0.0001, and SMD Chinese ethnicity=0.0009).

**Table 2:** General characteristics of myocardial infarction cases and controls.

Variable			Cases (n=2547)			Controls (n=2500)			Unadjusted *P* value	
**Demography**										
	Age (years), mean (SD)		50.3 (9.4)			48.9 (10)			Frequency-matched	
	Female sex, n (%)		223 (8.76)			222 (8.88)			Frequency-matched	
	**Major ethnicities, n (%)**									Frequency-matched
		Malay	1548	(60.77)	1536		(61.44)			
		Chinese	346	(13.58)	347		(13.88)			
		Indian	487	(19.12)	455		(18.20)			
		Sabah and Sarawak Bumiputera^a^	150	(5.89)	150		(6.00)			
		Orang Asli	2	(0.08)	1		(0.04)			
		Others	14	(0.55)	11		(0.44)			
**Tobacco use and tobacco alternatives, n (%)**										
	**Smoking^b^**									<.001^c^
		Never	604 (25)			1120 (45.94)				
		Ex	325 (13.45)			501 (20.55)				
		Current	1487 (61.55)			817 (33.51)				
	**Chewing tobacco**									.52
		Never	2328 (97.90)			2386 (98.31)				
		Ex	9 (0.38)			9 (0.37)				
		Current	41 (1.72)			32 (1.32)				
	**Vaping^b^, n (%)**									.21
		Yes	28 (1.18)			39 (1.61)				
		No	2343 (98.82)			2386 (98.39)				
	Number of cigarettes per day in current smokers		1475	20 (10-20)	806		10 (5-20)	<.001^c^		
	Number of chewing tobacco products per day in current users		38	2 (2-4)	31		2 (2-4)	.73		
	Number of times vaping per day in current users		23	3.5 (1-10)	36		5 (1.5-10)	.77		
**Consanguinity, n (%)**										
	Parents first cousins (yes)^b^		68 (2.86)			66 (2.72)			0.96	
	Spouse first cousin (yes)^b^		41 (1.76)			34 (1.43)			0.37	
**Conventional risk factors, n (%)**										
	History of high blood pressure—self-report *(Yes)*^b^		872 (35.68)			500 (20.44)			<.001^c^	
	History of diabetes mellitus—self-report *(Yes)*^b^		691 (28.40)			308 (12.59)			<.001^b^	
	Family history of CHD^d^—self-report *(Yes)*^b^		270 (11.36)			220 (9.02)			.007^c^	
	Waist-to-hip ratio		2108	0.96 (0.93-0.98)	2285		0.94 (0.90-0.97)	<.001^c^		
	BMI (kg/m^2^)		2258	26.4 (23.9-29.5)	2399		26.9 (24.0-30.0)	.013^c^		
**Sociodemographic, n (%)**										
	**Monthly personal income (Malaysian ringgit)^b^**									<.001^c^
		<1500 (US $ 357)	846 (37.58)			700 (30.24)				
		1500 to <3000 (US $ 357 to <714)	795 (35.32)			740 (31.96)				
		3000 to <4500 (US $ 714 to 1071)	342 (15.19)			440 (19.01)				
		4500 to <6000 (US $ 1071 to <1428)	159 (7.06)			226 (9.76)				
		6000 and above (US $ 1428 and above)	109 (4.84)			209 (9.03)				
	**Education level^b^**									<.001^c^
		None	63 (2.76)			34 (1.47)				
		Primary	358 (15.67)			234 (10.15)				
		Secondary	1260 (55.16)			1160 (50.30)				
		Higher secondary	284 (12.44)			276 (11.97)				
		Bachelors or diploma	275 (12.04)			517 (22.42)				
		Masters or higher	44 (1.93)			85 (3.69)				
	**Occupational group^b^**									<.001^c^
		Business or self-employed	516 (21.74)			483 (20.00)				
		Professional	526 (22.16)			785 (32.51)				
		Skilled labor	324 (13.65)			257 (10.64)				
		General labor	202 (8.51)			165 (6.83)				
		Farmer	70 (2.95)			53 (2.20)				
		Student	4 (0.17)			5 (0.22)				
		Housewife/house husband	84 (3.54)			77 (3.19)				
		Unemployed	111 (4.68)			109 (4.51)				
		Retired	219 (9.22)			273 (11.30)				
		Other	318 (13.40)			208 (8.61)				

^a^Sabah and Sarawak Bumiputera include Iban: Kadazan Dusun; Bidayuh: Melanau, other Bumiputera of Sabah, and other Bumiputera of Sarawak ethnicities.

^b^Smoking: information for smoking was available (missing %) in 2416 cases (5.1%) and 2438 controls (2.5%); chewing tobacco in 2378 cases (6.6%) and 2427 controls (2.9%); vaping in 2371 cases (6.9%) and 2386 controls (4.6%); marriage of a parent to a first cousin in 2376 cases (6.7%) and 2430 controls (2.8%); marriage to a spouse that is a first cousin in 2330 cases (8.5%) and 2385 controls (4.6%); history of high blood pressure in 2444 cases (4%) and 2446 controls (2.2%); history of diabetes in 2433 cases (4.5%) and 2446 controls (2.2%); family history of CHD in 2376 cases (6.7%) and 2440 controls (2.4%); monthly income level in 2251 cases (11.6%) and 2315 controls (7.4%); education level in 2284 cases (10.3%) and 2306 controls (7.8%); occupation in 2374 cases (6.8%) and 2415 controls (3.4%). Normally distributed variables are presented as mean (SD), and nonnormally distributed variables are presented as median (IQR), and categorical variables are presented as n (%).

^c^*P*<.05 was calculated from the unadjusted *χ*^2^ test of independence or Fisher exact test (n≤5 in any cell) for categorical variables and from *t* test for equalities of the means or Mann-Whitney *U* test (nonnormally distributed data) for continuous variables.

^d^CHD: coronary heart disease.

Approximately 61.55% (1487/2416) of MI cases were current smokers, compared with only 33.51% (817/2438) of controls (*P*<.001; Table 2). Cigarette consumption in smokers was higher among cases than in controls (median, IQR daily consumption for cases vs controls 20, 10-20 *v*s 10, 5-20) cigarettes per day; *P*<.001). However, the prevalence of tobacco consumption by chewing and vaping was low and did not materially differ among cases and controls. Other risk factors that had a higher prevalence in MI cases than controls included high blood pressure (872/2444, 35.68% vs 500/2446, 20.44%; *P*<.001; Table 2), diabetes mellitus (691/2433, 28.4% vs 308/2446, 12.59%; *P*<.001), and family history of CHD (70/2376, 11.36% vs 220/2440, 9.02%; *P*=.007). MI cases also had higher median levels of waist-to-hip ratio than controls (0.96 vs. 0.94; *P*<.001), but median levels of BMI were not materially different.

Indicators of lower socioeconomic status were more common among MI cases than controls, including higher percentages with a monthly income below MYR1500 (US $357; 846/2251, 37.58% cases vs 700/2315, 30.24% controls; *P*<.001), and educational attainment to primary school level or lower (421/2284, 18.43% vs 268/2306, 11.62%; *P*<.001). The prevalence of participants reporting being the offspring of a first cousin marriage (68/2376, 2.86% vs 66/2430, 2.72%) or having a first cousin as a spouse (41/2330, 1.76% vs 34/2385, 1.43%) was not materially higher in cases than in controls. Additional details about the baseline distribution of characteristics, subdivided by age group and sex, are outlined in Multimedia Appendix 1, Tables S2 and S3.

Crude and adjusted (age and sex) logistic regression models for traditional risk factors are summarized in Table 3. Current smoking (OR 4.11, 95% CI 3.56-4.75; *P*<.001), previous smoking (OR 1.34, 95% CI 1.12-1.60; *P*=.001), history of high blood pressure (OR 2.13, 95% CI 1.86-2.44; *P*<.001), history of diabetes mellitus (OR 2.72, 95% CI 2.34-3.17; *P*<.001), family history of CHD (OR 1.28, 95% CI 1.07-1.55; *P*=.009), and obesity (BMI >30 kg/m^2^; OR 1.19, 95% CI 1.05-1.34; *P*<.01) were associated with MI in age- and sex-adjusted models.

**Table 3:** Odds ratios for established myocardial infarction risk factors.

Risk factor			Crude odds ratio (95% CI)		Adjusted odds ratio for age and sex (95% CI)
Smoking cigarettes					
	Ex	1.19 (1.00-1.41)^a^		1.34 (1.12-1.60)^a^	
	Current	3.33 (2.93-3.79)^b^		4.11 (3.56-4.75)^b^	
History of high blood pressure			2.17 (1.91-2.46)^b^		2.13 (1.86-2.44)^b^
History of diabetes mellitus			2.74 (2.37-3.19)^b^		2.72 (2.34-3.17)^b^
Family history of CHD^c^			1.28 (1.06-1.54)^d^		1.28 (1.07-1.55)^d^
Obesity (BMI >30 kg/m^2^)			1.16 (1.03-1.31)^a^		1.19 (1.05-1.34)^d^

^a^*P*<.05.

^b^*P*<.001.

^c^CHD: coronary heart disease.

^d^*P*<.01.

## Discussion

Although Malaysia is experiencing a substantial increase in the burden of CVD, the risk factors for CHD in this population have been relatively little studied. We established the MAVERIK study, an epidemiological bioresource comprising over 2500 confirmed cases of acute MI and 2500 controls, and confirmed the importance of several known risk factors for MI (eg, tobacco consumption, history of diabetes, and hypertension). To our knowledge, this represents the largest case-control study of MI and related traits in Malaysia. Our long-term objective is to enable direct investigation of genetic and other determinants of MI in this multi-ethnic population. In this paper, we report the design and initial results of this study, which suggest several conclusions.

First, our study has demonstrated the ability to use modern and standardized epidemiological methods in a national study in Malaysia encompassing 17 hospitals. For example, the study used electronic data collection, including apps with *intelligent* study forms, and collected several types of biological samples, including serum, plasma, DNA, and nails.

Second, the mean age at first confirmed MI was only 50 years in Malaysia, suggesting that premature CHD is highly prevalent in this population.

Third, this study’s multi-ethnic population broadly mirrors the overall ethnic composition of the Malaysian national population, which is approximately 61.8% Malay, 21.4% Chinese, 6.4% Indian, and 0.9% others and 9.6% non-Malaysian resident [20]. Malaysia’s complex genetic ancestry is distinct from the commonly studied East Asian populations (eg, Chinese and Japanese) and other Southeast Asian populations (eg, Thai and Filipino) [21]. In contrast, most existing genetic studies of MI and cardiometabolic risk factors involve European or East Asian (eg, Chinese and Japanese) descent populations. The distinctive genetic architecture of the Malaysian population suggests that, with the use of genome sequencing technologies, the MAVERIK study should help identify population-specific genetic risk factors, leveraging transethnic differences. For example, variants in the myosin binding protein C3 (*MYBPC3*) gene region, which are common (2%-8%) in South Asian ancestry populations but rare (<0.1%) in European ancestry populations, are associated with an increased risk of hypertrophic cardiomyopathies [22].

Fourth, an analysis of initial baseline data supports the validity of the MI outcomes recorded, suggested by the observation of expected associations of MI risk with a panel of conventional risk factors, including high blood pressure, diabetes mellitus, family history of coronary disease, and higher waist-to-hip ratio (a marker of visceral adiposity). Finally, the study’s initial data suggest that in Malaysia, a middle-income country with a population of 32 million people in the midst of economic and epidemiological transition, there are inverse associations of indicators of socioeconomic status (such as income and educational attainment) with MI risk, a pattern similar to that observed in many high-income populations in Western countries.

The strengths and potential limitations of the MAVERIK study merit consideration. Retrospective case-control studies of MI can usefully complement prospective studies because the former involves the ascertainment of exposure information and the blood sampling of people who have already developed MI and a comparable group of controls without MI, enabling the rapid and cost-effective accrual of large numbers of relevant cases. By contrast, prospective studies require many tens of thousands of people to be screened (with all of their blood samples kept in frozen storage) and followed for several years to accrue a sizable number of MI cases. Retrospective studies are also often able to include large numbers of individuals who have developed the disease at younger ages, when associations with risk factors are often stronger, providing particularly sensitive tests of certain hypotheses. Furthermore, owing to the richness of data collected, the MAVERIK study allows for the use of a range of analytical techniques, including machine learning approaches [23,24].

As demonstrated by the Wellcome Trust Case-Control Consortium [11], Myocardial Infarction Genetics Consortium [25], Pakistan Risk of Myocardial Infarction Study [26], and Bangladesh Risk of Acute Cardiovascular Events study [13], case-control studies can powerfully and efficiently facilitate genetic discovery and can use genomic data to quantify and robustly correct for any population structure. Furthermore, the International Study of Infarct Survival (ISIS) (United Kingdom–based) [27] and INTERHEART (conducted across 52 countries) studies [28] have demonstrated that appropriately conducted, large case-control studies can usefully address some nongenetic hypotheses in MI, such as tobacco and alcohol consumption, and serological evidence of infection. However, INTERHEART did not focus extensively on Malaysia; INTERHEART-Malaysia involved approximately only 100 MI cases [29].

Nevertheless, particularly regarding nongenetic hypotheses, retrospective case-control studies may be liable to potential biases, such as selection and recall biases. To minimize such biases, standardized questioning approaches were used for all study participants, and both cases and controls were selected from the same population. Furthermore, there may be less scope for recall bias in a study of acute MI than in a study of chronic stable CHD, as hours rather than months or years may have elapsed since the index event. As noted earlier, the selection of controls in case-control studies invariably involves trade-offs between scientific rigor and feasibility. Following the examples of INTERHEART [28], Pakistan Risk of Myocardial Infarction Study [26], and Bangladesh Risk of Acute Cardiovascular Events study [13], we chose to recruit controls drawn from attendants of people visiting outpatient clinics or (nonblood-related) attendants of cardiac patients. Studies of plasma components may be affected by case-control studies of acute MI because certain circulating markers may be altered by the sampling of blood within 24 hours of MI symptoms. If, as in MAVERIK, the time since the onset of symptoms has been recorded, any material bias can be quantified and at least partially corrected for. Similar considerations apply to fasting status and the recording of the time since the last meal. We seek to evaluate the findings of the MAVERIK study with reference to prospective cohort studies in Malaysia (eg, the Malaysian Cohort project) [30]. Finally, it is important to recognize that we were unable to screen controls for silent MI and that the external generalizability of the MAVERIK study may be limited as the study comprises 91.18% of male participants.

In conclusion, the MAVERIK study is a large, multi-ethnic epidemiological resource for CHD that should help identify and evaluate genetic and other determinants of MI in Malaysia. It should help to hasten the discovery of disease-causing pathways and inform regionally appropriate strategies that optimize public health action.

## Appendix

Multimedia Appendix 1 Malaysian Acute Vascular Events Risk, a resource to study genetic and other determinants of first-ever myocardial infarction in Malaysia.

## Abbreviations

CHD: coronary heart disease
CVD: cardiovascular disease
IMR: Institute for Medical Research
MAVERIK: Malaysian Acute Vascular Events Risk
MI: myocardial infarction
OR: odds ratio
SMD: standardized mean difference
