# Supplementary material for: Investigating Genetic and Other Determinants of First-Onset Myocardial Infarction in Malaysia: Protocol for the Malaysian Acute Vascular Events Risk Study
Source: JMIR Res Protoc. 2022 Feb 10;11(2):e31885. doi: 10.2196/31885 (PMC8874931; doi:10.2196/31885)
Supplement: Multimedia Appendix 1 [file resprot_v11i2e31885_app1.docx]

**Multimedia Appendix 1**. Malaysian Acute Vascular Events Risk, a resource to study genetic and other determinants of first-ever myocardial infarction in Malaysia.

Supplementary materials to “Chowdhury *et al.* The Malaysian Acute Vascular Events Risk (MAVERIK) study: a resource to study genetic and other determinants of first-ever myocardial infarction in Malaysia.”

**MAVERIK Clinical Collaborators.** *Listed alphabetically on first name.*

Alfieyanto Syaripuddin^1^, Ang Kuan Hooi^1^, Barbara Anak Michael^2^, Chan Beng Zhong^2^, Che Ratna Shakila Johari^3^, Chia Chun Keat^1^, Chin Fah Shin^1^, Chu Chong Mow^1^, Chua Ping Lik^4^, Delarina Frimawati Othman Andu^2^, Emelyne Bani Jam^1^, Esmond Khoo Zhen Lin^5^, Farahdina Ramli^6^, Gan Fei Lee^2^, Grazele Jenarun^1^, Halimah Ishak^7^, Jamuna Radha Krishna^5^, Khaw Seong Kooi^1^, Khaw Yeong Mei^8^, Liyana Ahamad Fouzi^9^, Ma Kian Fung^1^, Mabelle Wong^1^, Marisa Khatijah Borhan^5^, Mohd Ediamin Suhaimi^1^, Muhammad Adlan Salleh^5^, Muhd Afifi Bahren^3^, Naimah Hamid^3^, Nida’ Ul-Huda Adznan^2^, Noraini Seman^9^, Noramalina Ramli^3^, Norazrulrizal Mat Noor^10^, Normalah Pungging^1^, Norsanisah Ahmad^3^, Norsima Nazifah Sidek^9^, Nur Asyikin Mohd Yunus^4^, Nurhasyimah Mohammad Sobri^3^, Ong Tiong Kiam^11^, Pradeep Kumar Nair Arumagam^7^, Punitha Kristummoonthy^2^, Richard Long Chay Shien^2^ , Rini Arwina Arshad^6^, Rohasmas Che Lah^3^, Ruhaiza Mohamad^2^, Sahrin Saharudin^1^, Saravanan Vengadesa Pillai^2^, Shalini Vijayasingham^2^, Shamila Sutharsan^2^, Sia Koon Ket^10^, Siti Salmah Ismail^3^, Syarifah Nurul Ain Syed Badaruddin^1^, Tan Chor Keng^1^, Tan Hui-Xin^2^, Teoh Eu Vin^1^, Ting Seng Kiat^2^, Toh Seng Hsiung^4^, Vijay Mukundadevan^7^, Vimala Veni Subramanian^1^, Viyaendran Rajalingam^5^, Yap Chee Jiek^7^, Yen Chia How^1^, Zariah Abdul Aziz^9^.

^1^ Hospital Queen Elizabeth II, Department of Cardiology & Clinical Research Centre, Sabah, Malaysia

^2^ Hospital Melaka, Department of General Medicine & Clinical Research Centre, Melaka, Malaysia

^3^ Hospital Pulau Pinang, Department of Cardiology, Pulau Pinang, Malaysia

^4^ Hospital Tengku Ampuan Rahimah, Department of General Medicine, Selangor, Malaysia

^5^ Hospital Sungai Buloh, Department of General Medicine, Selangor, Malaysia

^6^ Hospital Shah Alam, Department of General Medicine, Selangor, Malaysia

^7^ Hospital Kuala Lumpur, Department of General Medicine, Kuala Lumpur, Malaysia

^8^ Institute for Medical Research, Ministry of Health, Malaysia

^9^ Hospital Sultanah Nur Zahirah, Clinical Research Centre, Terengganu, Malaysia

^10^ Hospital Tuanku Fauziah, Department of General Medicine, Perlis, Malaysia

^11^ Pusat Jantung Sarawak, Department of Cardiology, Sarawak, Malaysia

| **Supplement Table 1** Dietary intake information collected in the MAVERIK study | |
| --- | --- |
| Food group | Key information recorded – including those reflecting local habits |
| Cereal and cereal products | White rice, flavoured rice (e.g. *nasi lemak, nasi dagang*), paratha (e.g. *roti telur*), noodles (e.g. *mee kuning, mihun*), pasta, bread |
| Fast foods | Burger, fried chicken, pizza, French fries, sausage/hotdog/frankfurter, nuggets |
| Meat and meat products | Chicken, meat, mutton, internal organs (e.g. liver, spleen, lungs), chicken/meat ball, ham, bacon, luncheon meat, pork |
| Fish and seafood | Marine fish, prawn, squid, crab, shellfish, crab balls or cake, crab crackers |
| Eggs | Any egg |
| Legumes and legume products | Soy products (eg. *taufufa, tauhu*), fermented soybeans (*tempe*), legumes (e.g. groundnuts, chickpeas) |
| Milk and milk products | Fresh milk, commercial milk, evaporated milk, yogurt, powdered milk, cheese |
| Vegetables | Leafy green vegetables, legume vegetables (e.g. ladies’ fingers, string bean), tuber vegetables (e.g. yam), fruit vegetables (e.g. luffa, baby corn), cabbages (e.g. cabbage, cauliflower), local fresh salads (*ulam-ulaman*) |
| Fruits | Any fruit |
| Drinks and alcoholic drinks | Sugar-sweetened beverages, alcoholic beverages (e.g. *todi*, beer, shandy), cordial syrup, yogurt drinks, coffee, tea, water, chocolate/malted milk |
| Confectionaries | Local desserts (*kuih*), sweets (e.g. lolly ice), cake, pastries, snacks/crackers |
| Bread spread | Jam, *kaya*, butter, peanut butter, cream cheese, chocolate spread, margarine |
| Flavours | Sugar, honey, condensed milk, condiment, shrimp paste/*belacan*, salad dressing |
| Others | Soya bean oil, sunflower oil, olive oil, palm oil, sugar, salt, coconut milk |

**Supplement Figure 1** Summary of procedures for processing, transportation, and storage of MAVERIK biological samples. IMR; Institute for Medical Research Malaysia.


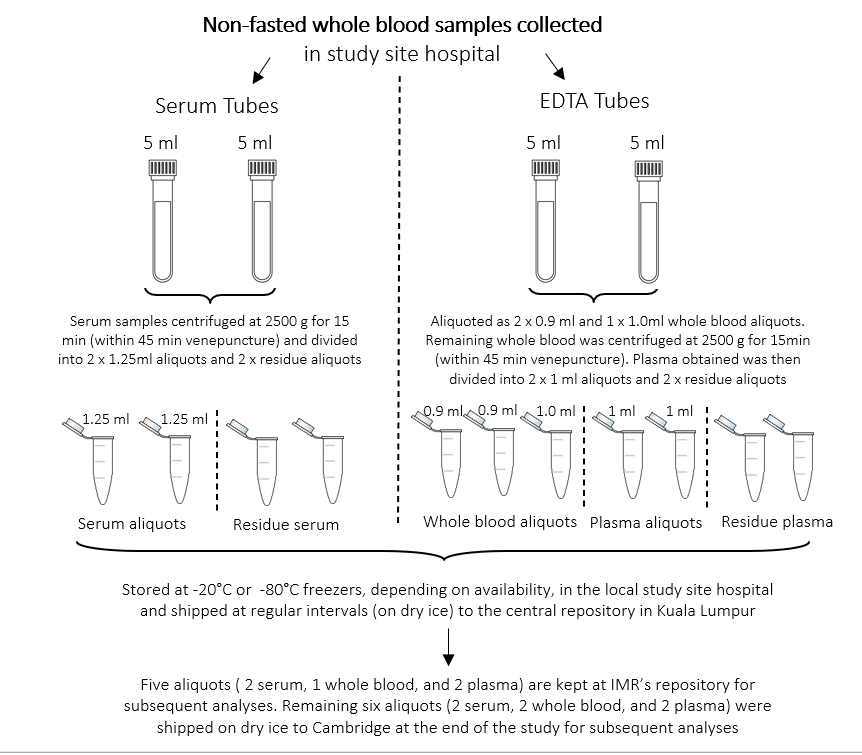


**Supplement Figure 2** Unadjusted prevalence of selected baseline variables by age and status. (a) Self-reported history of diabetes; (b) Current smokers; (c) Self-reported history of high blood pressure; (d) Overweight/obese (BMI>25kg/m^2^)


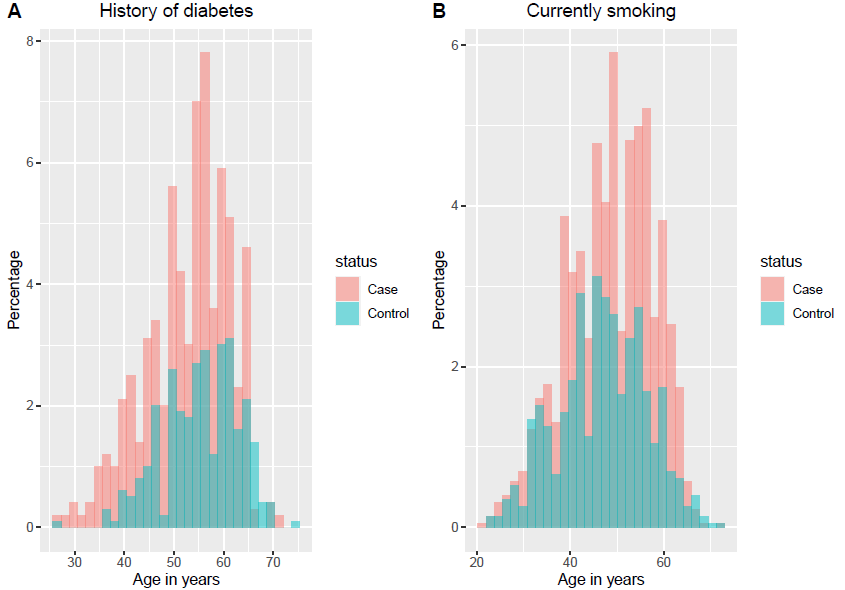


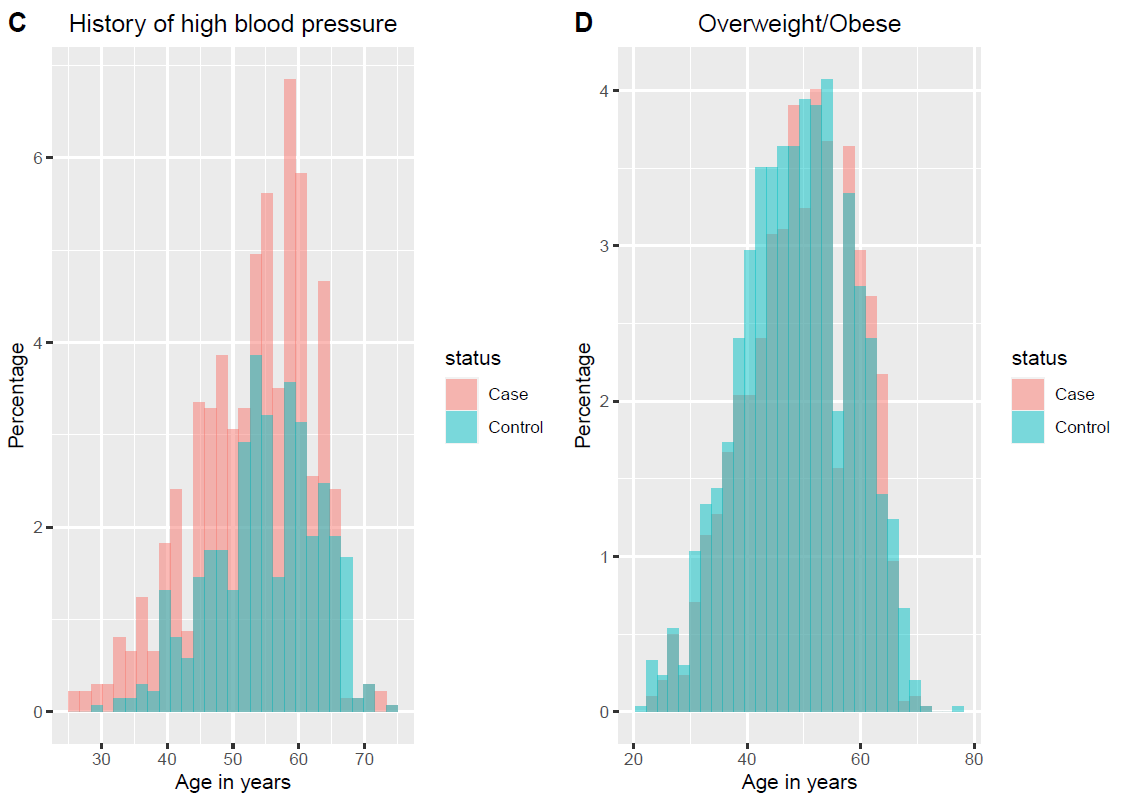


**Supplement Figure 3** Distribution of continuous variables by status. (a) Age; (b) Body Mass Index (BMI); (c)

Waist-to-hip ratio; (d) Weight; (e) Height; (f) Hips; (g) Waist


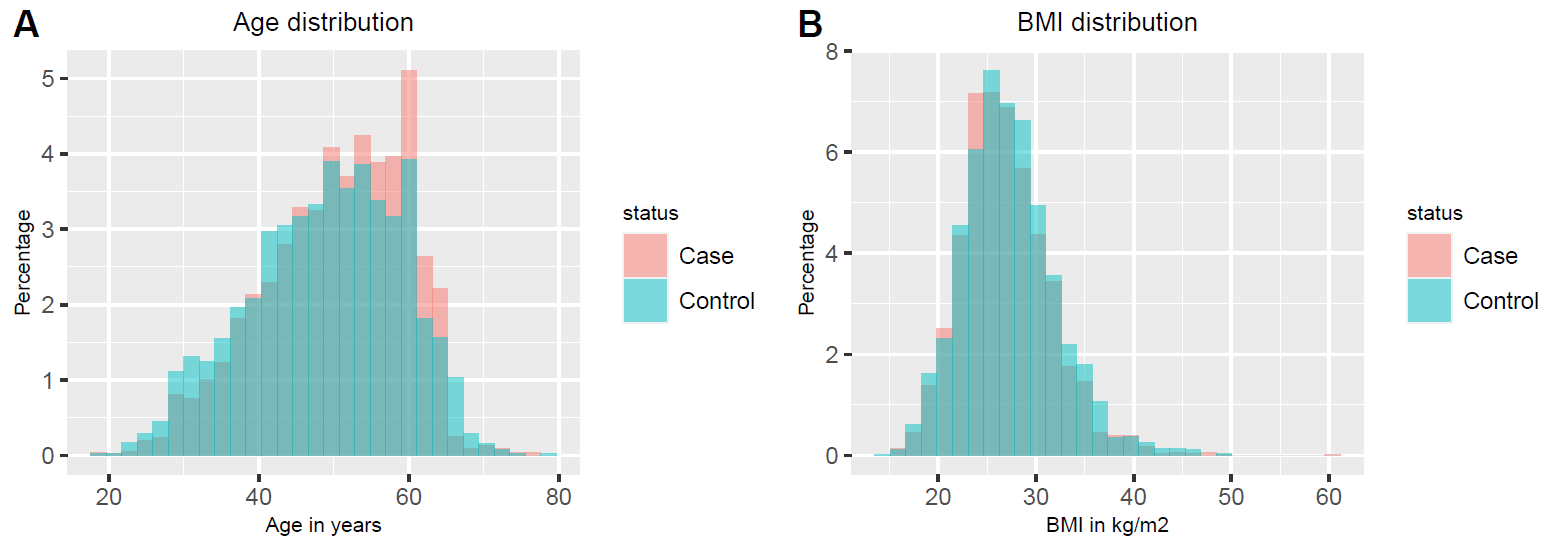


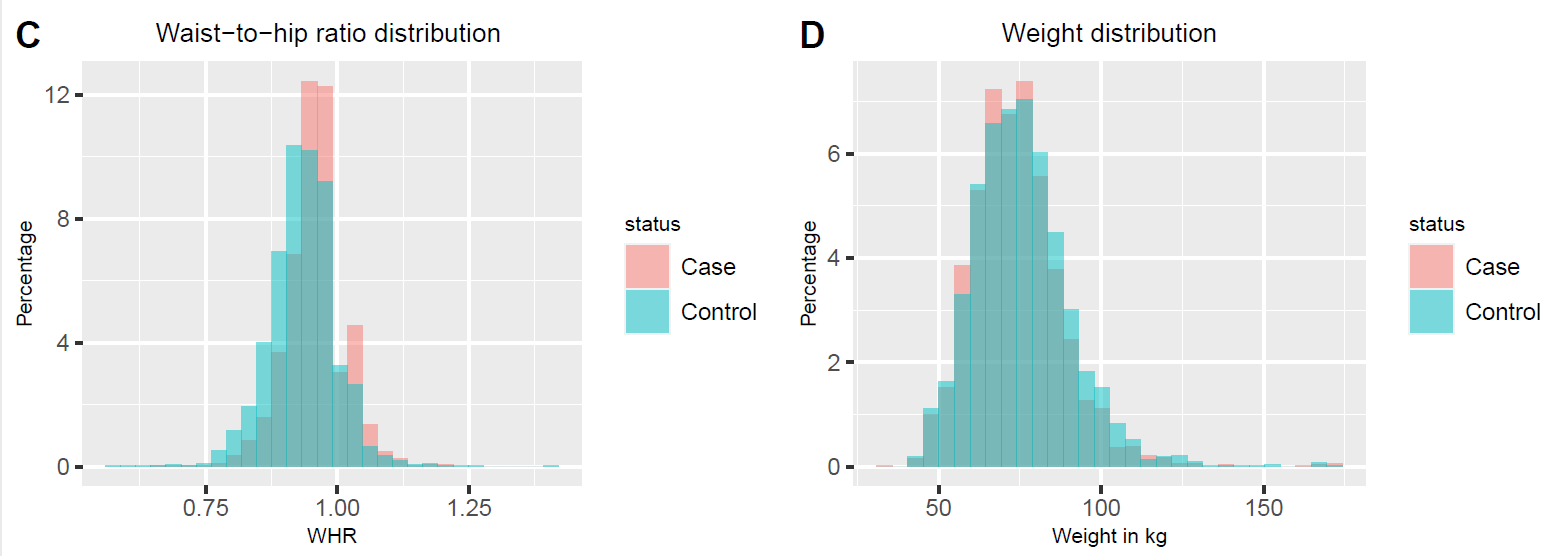


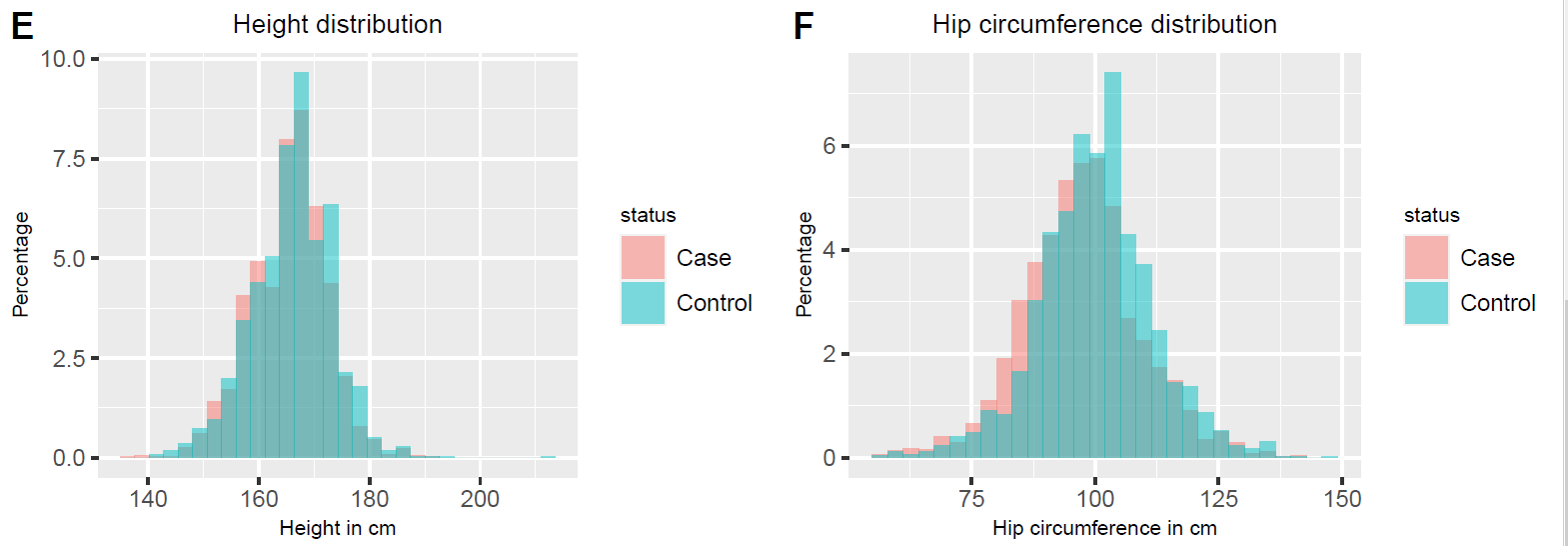


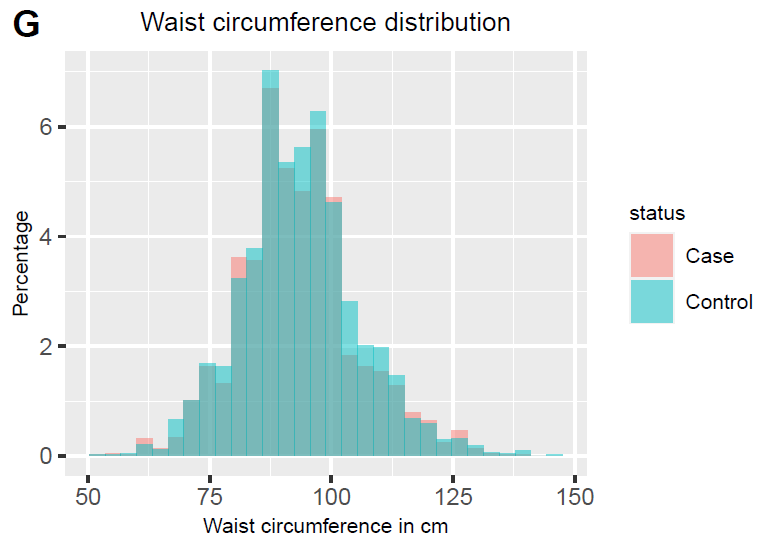


| **Supplement Table 2** Summary characteristics by age and sex in controls (n=2,500) | | | | | | | | | | |
| --- | --- | --- | --- | --- | --- | --- | --- | --- | --- | --- |
|  | **Age group** | | | | | **Sex** | | | | |
|  | *≤ 50 years old* | | *> 50 years old* | |  | *Female* | | *Male* | |  |
| Number of control participants | 1,345 | | 1,155 | |  | 222 | | 2,278 | |  |
| Variable | *N* | *Mean (SD) or %* | *N* | *Mean (SD) or %* | *P-value* | *N* | *Mean (SD) or %* | *N* | *Mean (SD) or %* | *P-value* |
| ***Demography*** | | | | | | | | | | |
| Age | 1,345 | 43 (37-47) | 1,155 | 57 (54-61) | *-* | 222 | 53 (47-60) | 2278 | 49 (42-56) | <0.0001* |
| Female Sex | 87 | 6.5*%* | 135 | 11.7*%* | <0.0001* | 222 | 100% | 0 | 0% | - |
| Major Ethnicities  *Malay*  *Chinese*  *Indian*  *Sabah & Sarawak Bumiputera ^●^*  *Orang Asli*  *Others* | 865  144  235  96  1  4 | 64.3*%*  10.7*%*  17.5*%*  7.1*%*  0.1*%*  0.3*%* | 671  203  220  54  0  7 | 58.1*%*  17.6*%*  19.1*%*  4.7*%*  0*%*  0.6*%* | <0.0001* | 127  22  60  11  0  2 | 57.2*%*  9.9*%*  27.0*%*  5.0*%*  0.0*%*  0.9*%* | 1,409  325  395  139  1  9 | 61.9*%*  14.3*%*  17.3*%*  6.1*%*  0.0*%*  0.4*%* | 0.007* |
| ***Tobacco use & tobacco alternatives***  Smoking ^a^  *Never*  *Ex*  *Current*  Chewing tobacco^a^  *Never*  *Ex*  *Current*  Vaping ^a^  *Yes*  *No*  No. of cigarettes per day in smokers  No. of smokeless products per day in users  No. of times vaping per day in users | 562  248  509  1,297  5  15  36  1,279  501  15  33 | 42.6*%*  18.8*%*  38.6*%*  98.5*%*  0.4*%*  1.1*%*  2.7*%*  97.3*%*  10 (6-20)  2 (1-6)  5 (1-10) | 558  253  308  1,089  4  17  3  1,107  305  16  3 | 49.9*%*  22.6*%*  27.5*%*  98.1*%*  0.4*%*  1.5*%*  0.3*%*  99.7*%*  10 (5-20)  3 (2-15)  10 (5-10) | <0.0001*  0.67  <0.0001*  0.49  0.74  0.66 | 202  5  10  208  1  5  1  213  10  5  1 | 93.1*%*  2.3*%*  4.6*%*  97.2*%*  0.5*%*  2.3 *%*  0.5*%*  99.5*%*  7.5 (1-20)  1 (1-1)  2 (2-2) | 918  496  807  2,178  8  27  38  2,173  796  26  35 | 41.3*%*  22.3*%*  36.3*%*  98.4*%*  0.4*%*  1.2*%*  1.7*%*  98.3*%*  5 (1-10)  2.5 (2-5)  10 (5-20) | <0.0001*  0.24  0.25  0.24  0.07  0.72 |
| ***Consanguinity*** | | | | | |  |  |  |  |  |
| Parent first cousin | 27 | 2.1*%* | 41 | 3.7*%* | 0.02* | 5 | 2.3*%* | 63 | 2.9*%* | 0.83 |
| Spouse first cousin | 12 | 0.9*%* | 22 | 2.0*%* | 0.03* | 1 | 0.5*%* | 33 | 1.5*%* | 0.36 |
| ***Conventional risk factors*** | | | | | | | | | | |
| History of high blood pressure (self-report) | 135 | 10.2*%* | 365 | 32.4*%* | <0.0001* | 71 | 32.7*%* | 429 | 19.3*%* | <0.0001* |
| History of diabetes mellitus (self-report) | 82 | 6.2*%* | 226 | 20.1*%* | <0.0001* | 48 | 22.1*%* | 260 | 11.7*%* | <0.0001* |
| Family history of CHD (self-report) | 118 | 8.9*%* | 102 | 9.1*%* | <0.0001* | 30 | 13.8*%* | 190 | 8.6*%* | 0.01* |
| Waist-to-hip ratio  Body Mass Index (BMI) | 1238  1305 | 0.93(0.89-0.97)  27.1(24-30.4) | 10471094 | 0.94(0.91-0.98)  26.7(24-29.5) | *<0.0001**  *0.04** | 203  212 | 0.92(0.86-0.96)  27.9(24.9-31.2) | 2,0822,187 | 0.94(0.9-1.0)  26.8(24-29.8) | <0.001*  0.002* |
| ***Socio-demographic indices*** | | | | | | | | | | |
| Monthly income (Malaysian ringgit)  *< 1500*  *1500-3000*  *3000-4500*  *4500-6000*  *>6000* | 291  439  280  126  127 | 23.0*%*  34.8*%*  22.2*%*  10.0*%*  10.1*%* | 409  301  160  100  82 | 38.9*%*  28.6*%*  15.2*%*  9.5*%*  7.8*%* | <0.0001* | 108  40  23  16  11 | 54.6*%*  20.2*%*  11.6*%*  8.1*%*  5.6*%* | 592  700  417  20  198 | 28.0*%*  33.1*%*  19.7*%*  9.9*%*  9.4*%* | <0.0001* |
| Education Level  *None*  *Primary*  *Secondary*  *Higher secondary*  *Bachelors or Diploma*  *Masters or higher* | 10  64  597  155  361  52 | 0.8*%*  5.2*%*  48.2*%*  12.5*%*  29.1*%*  4.2*%* | 24  170  563  121  156  33 | 2.3*%*  15.9*%*  52.8*%*  11.3*%*  14.6*%*  3.1*%* | <0.0001* | 13  33  99  18  40  3 | 6.3*%*  16.0*%*  48.1*%*  8.7*%*  19.4*%*  1.5*%* | 21  201  1,061  258  477  82 | 1.0*%*  9.6*%*  50.5*%*  12.3*%*  22.7*%*  3.9*%* | <0.0001* |
| Occupational Group  *Business or self-employed*  *Professional*  *Skilled labour*  *General labour*  *Farmer*  *Student*  *Housewife / house-husband*  *Unemployed*  *Retired*  *Other* | 280  516  174  104  25  4  25  43  23  122 | 21.5*%*  39.5*%*  13.3*%*  8.0*%*  1.9*%*  0.3*%*  1.9*%*  2.5*%*  1.8*%*  9.4*%* | 203  269  83  61  28  1  52  77  250  86 | 18.3*%*  24.2*%*  7.5*%*  5.5*%*  2.5*%*  0.1*%*  4.7*%*  6.9*%*  22.5*%*  7.8*%* | <0.0001* | 17  52  13  5  1  2  74  18  17  14 | 8.0*%*  24.4*%*  6.1*%*  2.4*%*  0.5*%*  0.9*%*  34.7*%*  8.5*%*  8.0*%*  6.6*%* | 466  733  244  160  52  3  3  91  256  194 | 21.2*%*  33.3*%*  11.1*%*  7.3*%*  2.4*%*  0.1*%*  0.1*%*  4.1*%*  11.6*%*  8.8*%* | <0.0001* |
| Normally distributed variables are presented as mean ± standard deviation (SD), not normally distributed variables are presented as median (IQR), categorical variables are presented as count (percentage). **p*-Values <0.05 are calculated from unadjusted Χ^2^ test of independence or Fisher’s Exact test (n≤5 in any cell) for categorical variable and from t-test for equalities of the means or Mann-Whitney U (non-normally distributed data) for continuous variables. *^●^* Sabah & Sarawak Bumiputera includes Iban, Kadazan, Dusun, Bidayuh, Melanau, other Bumiputera of Sabah and other Bumiputera of Sarawak ethnicities CHD: coronary heart disease; BMI: body mass index. Missingness ranged from 2.2% to 7.8%. | | | | | | | | | | |

| **Supplement Table 3** Summary characteristics by age and sex in cases (n=2,547) | | | | | | | | | | |
| --- | --- | --- | --- | --- | --- | --- | --- | --- | --- | --- |
|  | Age group | | | | | Sex | | | | |
|  | ≤ 50 years old | | > 50 years old | |  | Female | | Male | |  |
| Number of control participants | 1,213 | | 1,334 | |  | 223 | | 2,324 | |  |
| Variable | N | Mean (SD) or % | N | Mean (SD) or % | *P*-value | N | Mean (SD) or % | N | Mean (SD) or % | *P*-value |
| ***Demography*** | | | | | | | | | | |
| Age | 1,213 | 44 (38-48) | 1,334 | 57 (53-61) | - | 223 | 55 (49-61) | 2,324 | 51 (44-57) | <0.0001* |
| Female Sex | 72 | 5.9% | 151 | 11.3 | <0.0001* | 223 | 100% | 0 | 0% | - |
| Major Ethnicities  *Malay*  *Chinese*  *Indian*  *Sabah & Sarawak Bumiputera^●^*  *Orang Asli*  *Others* | 753  127  236  88  1  8 | 62.1%  10.5%  19.5%  7.3%  0.1%  0.7% | 795  219  251  62  1  6 | 59.6%  16.4%  18.8%  4.7%  0.1%  0.5% | <0.0001* | 130  20  62  11  0  0 | 58.3%  9.0%  27.8%  4.9%  0.0%  0.0% | 1,418  316  425  139  2  14 | 61.0%  14.0%  18.3%  6.0%  0.1%  0.6% | 0.012* |
| ***Tobacco use***  Smoking ^a^  *Never*  *Ex*  *Current*  Chewing tobacco^a^  *Never*  *Ex*  *Current*  Vaping ^a^  *Yes*  *No*  No. of cigarettes per day in smokers  No. of smokeless products per day in users  No. of times vaping per day in users | 223  119  819  1,123  3  21  20  1,125  812  19  17 | 19.2%  10.3%  70.5%  97.9%  0.3%  1.8%  1.8%  98.3%  20(10-20)  2(1-3)  3(1-6) | 381  206  668  1,205  6  20  8  1,218  663  19  7 | 30.4%  16.4%  53.2%  97.9%  0.5%  1.6%  0.7%  99.4%  18(10-20)  2(2-6)  10(1-10) | <0.0001*  0.66  0.014  0.65  0.27  0.44 | 185  8  17  203  2  3  0  207  17  3  0 | 88.1%  3.8%  8.1%  97.6%  1%  1.4%  0%  100%  12(10-20)  1(1-50)  - | 419  317  1,470  2,125  7  38  28  2,136  1,458  35  24 | 19%  14.4%  66.6%  97.9%  0.3%  1.8%  1.3%  98.7%  20(10-20)  2(2-4)  3.5(1-10) | <0.0001*  0.28  0.08  0.16  0.63  - |
| ***Consanguinity*** | | | | | | | | | | |
| Parent first cousin | 28 | 2.4% | 38 | 3.1% | 0.3 | 4 | 2.0% | 62 | 2.9% | 0.66 |
| Spouse first cousin | 14 | 1.2% | 27 | 2.2% | 0.07 | 2 | 1.0% | 39 | 1.8% | 0.58 |
| ***Conventional risk factors*** | | | | | | | | | | |
| History of high blood pressure (self-report) | 316 | 27.0% | 556 | 43.6% | <0.0001* | 131 | 61.8% | 741 | 33.2% | <0.0001* |
| History of diabetes mellitus (self-report) | 247 | 21.1% | 444 | 34.9% | <0.0001* | 118 | 55.4% | 573 | 25.7% | <0.0001* |
| Family history of CHD (self-report) | 138 | 12.1% | 132 | 10.7% | 0.29 | 24 | 11.5% | 246 | 11.4% | 0.93 |
| Waist-to-hip ratio  Body Mass Index (BMI) | 1,0211,092 | 0.96(0.93-0.98)  26.8(24.1-30.2) | 1087 1166 | 0.96(0.93-0.99)  26.0(23.7-29) | 0.11*  <0.001* | 187 195 | 0.95(0.89-0.98)  27.3(25-30.8) | 1,9212,063 | 0.96(0.93-0.98)  26.4(23.8-29.4) | 0.001*  0.002* |
| ***Socio-demographic indices*** | | | | | | | | | | |
| Monthly income (Malaysian ringgit)  *< 1500*  *1500-3000*  *3000-4500*  *4500-6000*  *>6000* | 325  440  181  94  59 | 29.6%  40.0%  16.5%  8.6%  5.4% | 521  355  161  65  50 | 45.2%  30.8%  14.0%  5.6%  4.3% | <0.0001* | 115  43  13  7  10 | 61.2%  22.9%  6.9%  3.7%  5.3% | 731  752  329  152  99 | 35.4%  36.5%  16.0%  7.4%  4.8% | <0.0001* |
| Education Level  *None*  *Primary*  *Secondary*  *Higher secondary*  *Bachelors or Diploma*  *Masters or higher* | 16  104  629  155  178  20 | 1.5%  9.4%  57.1%  14.1%  16.2%  1.8% | 47  254  631  129  97  24 | 4.0%  21.5%  53.4%  10.9%  8.2%  2.0% | <0.0001* | 18  50  93  11  18  6 | 9.2%  25.5%  47.5%  5.6%  9.2%  3.1% | 45  308  1,167  273  257  38 | 2.2%  14.8%  55.9%  13.1%  12.3%  1.8% | <0.0001* |
| Occupational Group  *Business or self-employed*  *Professional*  *Skilled labour*  *General labour*  *Farmer*  *Student*  *Housewife / house-husband*  *Unemployed*  *Retired*  *Other* | 256  313  205  107  21  4  19  28  22  163 | 22.5%  27.5%  18.0%  9.4%  1.9%  0.4%  1.7%  2.5%  1.9%  14.3% | 260  213  119  95  49  0  65  83  197  155 | 21.0%  17.2%  9.6%  7.7%  4.0%  0.0%  5.3%  6.7%  15.9%  12.5% | <0.0001* | 23  33  14  5  3  0  82  18  10  18 | 11.2%  16.0%  6.8%  2.4%  1.5%  0.0%  39.8%  8.7%  4.8%  8.7% | 493  493  310  197  67  4  2  93  209  300 | 22.7%  22.7%  14.3%  9.1%  3.1%  0.2%  0.1%  4.3%  9.6%  13.8% | <0.0001* |
| Normally distributed variables are presented as mean ± standard deviation (SD), not normally distributed variables are presented as median (IQR), categorical variables are presented as count (percentage). **p*-Values <0.05 are calculated from unadjusted Χ^2^ test of independence or Fisher’s Exact test (n≤5 in any cell) for categorical variable and from t-test for equalities of the means or Mann-Whitney U (non-normally distributed data) for continuous variables. *^●^* Sabah & Sarawak Bumiputera includes Iban, Kadazan, Dusun, Bidayuh, Melanau, other Bumiputera of Sabah and other Bumiputera of Sarawak ethnicities CHD: coronary heart disease; BMI: body mass index. Missingness ranged from 5.1% to 11.6%. | | | | | | | | | | |

**Supplement Table 4** STROBE Statement—Checklist of items that should be included in reports of case-control studies

|  | **Item No** | **Recommendation** | **Page** |
| --- | --- | --- | --- |
| **Title and abstract** | 1 | (*a*) Indicate the study’s design with a commonly used term in the title or the abstract | 1 |
|  |  | (*b*) Provide in the abstract an informative and balanced summary of what was done and what was found | 3 |
| *Introduction* | | |  |
| Background/rationale | 2 | Explain the scientific background and rationale for the investigation being reported | 4 |
| Objectives | 3 | State specific objectives, including any prespecified hypotheses | 4 |
| *Methods* | | |  |
| Study design | 4 | Present key elements of study design early in the paper | 4,5 |
| Setting | 5 | Describe the setting, locations, and relevant dates, including periods of recruitment, exposure, follow-up, and data collection | 5-9 |
| Participants | 6 | (*a*) Give the eligibility criteria, and the sources and methods of case ascertainment and control selection. Give the rationale for the choice of cases and controls | 5 |
|  |  | (*b*) For matched studies, give matching criteria and the number of controls per case | 5 |
| Variables | 7 | Clearly define all outcomes, exposures, predictors, potential confounders, and effect modifiers. Give diagnostic criteria, if applicable | 5-9 |
| Data sources/ measurement | 8* | For each variable of interest, give sources of data and details of methods of assessment (measurement). Describe comparability of assessment methods if there is more than one group | 5-9 |
| Bias | 9 | Describe any efforts to address potential sources of bias | 5 |
| Study size | 10 | Explain how the study size was arrived at | 5 |
| Quantitative variables | 11 | Explain how quantitative variables were handled in the analyses. If applicable, describe which groupings were chosen and why | 7, 12 |
| Statistical methods | 12 | (*a*) Describe all statistical methods, including those used to control for confounding | 8 |
|  |  | (*b*) Describe any methods used to examine subgroups and interactions | N.A. |
|  |  | (*c*) Explain how missing data were addressed | 8 |
|  |  | (*d*) If applicable, explain how matching of cases and controls was addressed | 8 |
|  |  | (*e*) Describe any sensitivity analyses | 8-9 |
| *Results* | | |  |
| Participants | 13* | (a) Report numbers of individuals at each stage of study—eg numbers potentially eligible, examined for eligibility, confirmed eligible, included in the study, completing follow-up, and analysed | N.A. |
|  |  | (b) Give reasons for non-participation at each stage | N.A. |
|  |  | (c) Consider use of a flow diagram | Fig 2 |
| Descriptive data | 14* | (a) Give characteristics of study participants (eg demographic, clinical, social) and information on exposures and potential confounders | Table 2  Supp Table 2-3 |
|  |  | (b) Indicate number of participants with missing data for each variable of interest | Table 2 |
| Outcome data | 15* | Report numbers in each exposure category, or summary measures of exposure | Table 2 |
| Main results | 16 | (*a*) Give unadjusted estimates and, if applicable, confounder-adjusted estimates and their precision (eg, 95% confidence interval). Make clear which confounders were adjusted for and why they were included | Table 3 |
|  |  | (*b*) Report category boundaries when continuous variables were categorized | Table 2 |
|  |  | (*c*) If relevant, consider translating estimates of relative risk into absolute risk for a meaningful time period | N.A. |
| Other analyses | 17 | Report other analyses done—eg analyses of subgroups and interactions, and sensitivity analyses | Supp Table 2-3 |
| *Discussion* |  |  |  |
| Key results | 18 | Summarise key results with reference to study objectives | 10 |
| Limitations | 19 | Discuss limitations of the study, taking into account sources of potential bias or imprecision. Discuss both direction and magnitude of any potential bias | 10-13 |
| Interpretation | 20 | Give a cautious overall interpretation of results considering objectives, limitations, multiplicity of analyses, results from similar studies, and other relevant evidence | 10-13 |
| Generalisability | 21 | Discuss the generalisability (external validity) of the study results | 10-13 |
| Other information |  |  |  |
| Funding | 22 | Give the source of funding and the role of the funders for the present study and, if applicable, for the original study on which the present article is based | 15 |

**ID: |___|: |___|___|: |___|___|___|___|**

**A/B** **Hospital ID** **Subject ID**

**(case/control)**

**Malaysia Acute Vascular Events RIsK (MAVERIK) study**

A case-control study of early onset acute coronary events in Malaysia

**Section 1: Administrative details**

| **Optional consent form signed? Yes / No**  **Name of the Subject:**  Title______________________    First name______________________  Middle name(s) ______________________  Surname______________________  **Address of the subject:**  Address 1______________________  Address 2______________________  Address 3 ______________________  City______________________  Postcode______________________  State______________________  Country ______________________  Contact number of the subject: ______________________  Name of the Hospital: _____________________________  Ward no/ OPD:___________________________________  National identification number:______________________  First interviewer name/ code:________________\|___\|___\|  Sections filled 1 2 3 4 5 6 7 8 9 10 11 12 | Date of enrolment: **\|___\|___\| \|___\|___\| \|___\|___\|___\|___\|**  day month year  Date of interview: **\|___\|___\| \|___\|___\| \|___\|___\|___\|___\|**  day month year  Start time: **\|___\|___\| : \|___\|___\|** AM/PM  End time: **\|___\|___\| : \|___\|___\|** AM/PM  End date: **\|___\|___\| \|___\|___\| \|___\|___\|___\|___\|**  day month year  Date of sampling: **\|___\|___\| \|___\|___\| \|___\|___\|___\|___\|**  day month year  Time of sampling: **\|___\|___\| : \|___\|___\|** AM/PM  Time of last meal: **\|___\|___\| : \|___\|___\|** AM/PM  Date of last meal: **\|___\|___\| \|___\|___\| \|___\|___\|___\|___\|**  day month year  Spare labels SP1 –4 used? Yes / No (please circle)  If Yes, complete sample tube code for each spare label used:  SP1 _____________________  SP2 _____________________  SP3 _____________________  SP4 _____________________  2nd interviewer name/code:____________________ \|___\|___\|  Sections filled 1 2 3 4 5 6 7 8 9 10 11 12 |
| --- | --- |

**Section 1 Complete? Yes**

**Section 2: Demographics**

| **SL No.** | **Question** | **Code** | **Response/ Code** |
| --- | --- | --- | --- |
| D1. | Source of information | Participant 1  Attendant 2 |  |
| D2. | Gender | Male 1  Female 2 |  |
| D3. | Date of birth | \|___\|___\| \|___\|___\| \|___\|___\|___\|___\|  Day Month Year |  |
| D4. | Age | Years (by current date) |  |
| D5 | Are you married? | Yes 1  No 2  Divorced/widowed 3 |  |
| D6. | Religion | Islam 1  Hinduism 2  Christianity 3  Buddhism 4  Others (Specify) 5 |  |
| D7. | Usual place of residence | Wilayah Persekutuan Kuala Lumpur 1  Wilayah Persekutuan Putrajaya 2  Johor 3  Kedah 4  Kelantan 5  Melaka 6  Negeri Sembilan 7  Pahang 8  Pulau Pinang 9  Perak 10  Perlis 11  Selangor 12  Terengganu 13  Sabah & Labuan 14  Sarawak 15  Other 16 |  |
| D8. | Ethnicity | Malay 1  Chinese 2  Indian 3  Serani 4  Iban 5  Kadazan 6  Dusun 7  Bidayuh 8  Melanau --9  Other Bumiputera of Sabah 10  Other Bumiputera of Sarawak 11  Orang Asli ------------------------------------------------12  Others, specify 13 |  |

**Section 2 Complete? Yes**

**Section 3: Socio-demographic information**

| **SL No.** | **Question** | **Code** | **Response/ Code** |
| --- | --- | --- | --- |
| SD1. | Number of total years of formal education (Select only one) | <1 1  1-5 2  6-12 3  >12 4 |  |
| SD2. | Highest level of education achieved by the participant (Select only one) | None 1  Primary 2  Secondary 3  Higher secondary 4  Bachelors or Diploma 5  Masters or higher 6 |  |
| SD3. | Which category best describes participant’s main occupation (select only one) | Business or self-employed 1  Professional (eg, office executive,  government or NGO staff) 2  Skilled labour (eg, industry, garments) 3  General labour (eg, building  construction) 4  Farmer 5  Student 6  Housewife/house-husband 7  Unemployed 8  Retired 9  Other (please specify) 10 |  |
| SD4. | Which category best represents the average monthly income of the household? | Less than RM 1500 1  RM 1500 to less than RM3000 2  RM 3000 to less than RM4500 3  RM 4500 to less than RM6000 4  RM 6000 and above 5 |  |
| SD5. | Which category best represents the average monthly income of the participant? | Less than RM 1500 1  RM 1500 to less than RM3000 2  RM 3000 to less than RM4500 3  RM 4500 to less than RM6000 4  RM 6000 and above 5 |  |
| SD6. | Is your father related to your mother? | Yes 1  No (Go to SD8)2 |  |
| SD7. | If yes, please specify how they are related? | 1^st^ cousin 1  2^nd^ cousin 2  Niece/nephew 3  Other relative, please specify 4 |  |
| SD8. | Are you married to someone you are related to? | Yes 1  No (Go to SD10)2 |  |
| SD9. | If yes please specify how you are related | 1^st^ cousin 1  2^nd^ cousin 2  Niece/nephew 3  Other relative, please specify 4 |  |
| SD10. | Where does participant currently live? | City 1  Rural 2 |  |
| SD11. | Imagine a scale of 1 to 10, where 1 is for the poorest people, and 10 is for the richest people in Malaysia. Indicate where on this scale you would place yourself now? | 1 2 3 4 5  6 7 8 9 10 |  |

**Section 3 Complete? Yes**

**Section 4: Signs and Symptoms (Only for Cases)**

| **SL No.** | **Question** | **Code** | **Response/ Code** |
| --- | --- | --- | --- |
| SSO1. | Onset of symptoms | \|___\|___\| \|___\|___\| \|___\|___\|___\|___\|  Day Month Year  \|___\|___\| : \|___\|___\| AM/PM |  |
| SSO2. | Arrived at hospital | \|___\|___\| \|___\|___\| \|___\|___\|___\|___\|  Day Month Year  \|___\|___\| : \|___\|___\| AM/PM |  |
| SSO3. | First healthcare contact for current symptoms (Select only one) | Private physician clinic 1  Private physician at home 2  Other hospital/clinic 3  Current hospital 4  Pharmacist 5  Traditional healer 6  Other/not known 7 |  |
| SSO4. | Source of referral  (Select only one) | Self-referral 1  Primary physician 2  Walk-in/ambulance 3  Inter-hospital 4  Intra-hospital 5  Pharmacist 6  Traditional healer 7  Other/not known 8 |  |
| SSO5. | Reasons for delay  (Select only one) | Patient’s unawareness of symptoms 1  Family did not consider emergency 2  Transport problem 3  Delay in care (eg, went or called OPD/GP care rather than coming to hospital) 4  Sought alternative treatment (eg, pharmacist, traditional/complementary practitioner) 5  Financial factors 6  Referred from another hospital 7  Long distance from hospital 8  Not applicable 9 |  |

**Section 4 Complete? Yes**

**Section 5: Past medical history**

| *Did any doctor ever tell you that you have one or more of the following diseases?* | | Yes | No | Age when first diagnosed | Type (if applicable) | |
| --- | --- | --- | --- | --- | --- | --- |
| E1 | High blood pressure | 1 | 2 | \|___\|___\| years |  | |
| E2 | High blood cholesterol | 1 | 2 | \|___\|___\| years |  | |
| E3 | Diabetes Mellitus (excluding gestational diabetes) | 1 | 2 | \|___\|___\| years | Type 1 ………………………..1  Type 2 ………………………..2  Unknown…………………….3 |  |

**Section 5 Complete? Yes**

**Section 6: Family History**

| Have your parents ever had one or more of the following? | | Mother | | | Father | | |
| --- | --- | --- | --- | --- | --- | --- | --- |
|  |  | Yes | No | Age first diagnosed | Yes | No | Age first diagnosed |
| G1 | Coronary Heart Disease | 1 | 2 | \|___\|___\|___\| years | 1 | 2 | \|___\|___\|___\| years |
| G2 | Stroke/stroke-related paralysis | 1 | 2 | \|___\|___\|___\| years | 1 | 2 | \|___\|___\|___\| years |
| G3 | High blood pressure | 1 | 2 | \|___\|___\|___\| years | 1 | 2 | \|___\|___\|___\| years |
| G4 | Type 2 diabetes mellitus | 1 | 2 | \|___\|___\|___\| years | 1 | 2 | \|___\|___\|___\| years |
| G5 | Sudden cardiac death | 1 | 2 | \|___\|___\|___\| years | 1 | 2 | \|___\|___\|___\| years |
| G6 | Any cancer | 1 | 2 | \|___\|___\|___\| years | 1 | 2 | \|___\|___\|___\| years |

**Section 6 Complete? Yes**

**Section 7: Tobacco usage**

| **SL No.** | | **Question** | | **Code** | | **Response/ Code** | |
| --- | --- | --- | --- | --- | --- | --- | --- |
| I1 | | Have you ever smoked tobacco? | Yes………………………………………..............1 (go to I2)  No, never smoked……...…………………… 2 (go to I20) | | |  | |
| I2 | | How old were you when you first started smoking? | \|___\|___\| years | | |  | |
| I3 | | Do you currently smoke tobacco? | Yes………………………………………..............1 (go to I4)  No……………………………………………………..2 (go to I7) | | |  | |
| I4 | | What type of tobacco product do you mainly smoke NOW? | Manufactured cigarettes…………….……………………………1  Hand-rolled cigarettes..……………...…………………………...2  Cigars……………..……………...………………………………………..3  Pipes…………………………………………………………………………4  Shisha……………………………………………………………………….5  Kretek……………………………………………………………………….6  Bidis………………………………………………………………………….7  Other………………………………………………………………………..8 | | |  | |
| I5 | | About how many cigarettes or cigars do you smoke on average each day NOW? | \|___\|___\|___\| cigarettes or cigars per day | | |  | |
| I6 | | How often have you smoked tobacco in the past year? (go to I20) | Smoked on most or all days……………..….......………………….1  Smoked occasionally…………..…………......………………………..2 | | |  | |
| I7 | | How old were you when you last stopped smoking? | \|___\|___\| years | | |  | |
| I8 | | In your lifetime, have you smoked on a total of at least 100 occasions? | Yes……………………………….……………………1 go to I9  No………………………………...…………………..2 go to I13  Do not know………………………………………3 go to I13 | | |  | |
| I9 | | How often have you smoked tobacco in the PAST? | Smoked on most or all days……………..….......………………1  Smoked occasionally…………..…………......…………………….2 | | |  | |
| I10 | | What type of tobacco product did you mainly smoke in the PAST? | Manufactured cigarettes…………….……………………………1  Hand-rolled cigarettes..……………...…………………………...2  Cigars……………..……………...………………………………………..3  Pipes…………………………………………………………………………4  Shisha……………………………………………………………………….5  Kretek……………………………………………………………………….6  Bidis………………………………………………………………………….7  Other………………………………………………………………………..8 | | |  | |
| I11 | | About how many cigarettes or cigars did you smoke on average each day in the PAST? | \|___\|___\|___\| cigarettes/cigars per day | | | | |
| I12 | | In the time that you smoked in the PAST, did you ever stop for more than 6 months? | Yes……………………………….………………………1 (go to I13)  No………………………………...……………………..2 (go to I20)  Do not know………………………………………..3 | | |  | |
| **SL No.** | | **Question** | | **Code** | | **Response/ Code** | |
| I13 | | Why did you stop smoking? (You can select more than one answer) | Pregnancy………………………………………………………….. 1  Illness or ill health……………………………………………….2  Doctors’ advice………………………………………………….. 3  Health precaution / concern……………………………… 4  Financial reasons…………………………………………………5  Family reasons…………………………………………………….6  Other……………………………………………………………….....7 | | |  | |
| *CHEWABLE tobacco use* | | | | | | | |
| I20 | | Do you use chewing tobacco (*e.g. sireh/betel and tobacco*)? | | Yes, on most or all days…………………………………1 (*go to I22)*  Yes, only occasionally………………………………......2 (*go to I22)*  No, gave up…………………………………...………………3 (*go to I21)*  Never used..…………………………………...…………….4 (*go to I25)* | |  | |
| I21 | | How old were you when you stopped using chewing tobacco? *[fill in if Former user or go to next section]* | | \|___\|___\| years | |  | |
| I22 | | How many times do/did you use this on an average day? | | \|___\|___\| number | | | |
| I23 | | How old were you when you started chewing tobacco on most days? | | \|___\|___\| years | | | |
| I24 | | What type of chewing tobacco do/did you mainly use? | | Please specify | |  | |
| I25 | | Do you do vaping or use e-cigarettes? | | Yes…………………………………..1 (*go to I26)*  No………………………………......2 (*go to Section 8)* | |  | |
| I26 | | How often a day do you vape / do you use e-cigarettes? | | \|___\|___\| number | |  | |
| I27 | | For how many years have you vaped or used e-cigarettes? | | \|___\|___\| years | |  | |
| I28 | | Which type of vaping product do you use? | | Disposable E-cigarettes………………………………………………..1  2-piece cigarette model.………………………………………………2  2-piece “eGO”style Vape pens..…………………………………..3  3-piece “eGO”style Vape pens ……………………………………4  Modified (Mod) style advanced e-cigarettes………………….…………………………………………………5  Not known…………….…………………………………………………….6 | |  | |

**Section 7 Complete? Yes**

**Section 8: Physical activity**

| **PLEASE NOTE THE VIGOUROUS PHYSICAL ACTIVITY THAT YOU DO** | | | |
| --- | --- | --- | --- |
| **PA010** | In a typical week, when you are well, on how many days have you done vigorous physical activity (e.g. carry heavy weights, fill the earth, aerobic exercises or fast cycling and others) for at least 10 minutes at a time?  Days in a week  No vigorous physical  activity | **PA011** | How much time did you usually spend doing vigorous physical activity on one of those days?  minutes  do not know / not sure |
| **PLEASE NOTE THE MODERATE PHYSICAL ACTIVITY THAT YOU DO** | | | |
| **PA020** | In a typical week, when you are well, on how many days have you done moderate physical activity (e.g. carry light weights, mop the floor, or normal rate of cycling and others) for at least 10 minutes at a time? This does not include walking  Days in a week  No moderate physical  activity | **PA021** | How much time did you usually spend doing moderate physical activity on one of those days?  minutes  do not know / not sure |
| **PLEASE NOTE THE AMOUNT OF WALKING THAT YOU DO** | | | |
| **PA030** | In a typical week, when you are well, on how many days have you walked for at least 10 minutes at a time?  Days in a week  No walking | **PA031** | How much time did you usually spend walking on one of those days?  minutes    do not know / not sure |
| **PLEASE NOTE THE SEDENTARY ACTIVITY THAT YOU DO** | | | |
| **PA040** | In a typical week, when you are well how much time did you spend **sitting** on a **week day**. Include time spent at work, at home, and in your free time, This may include time spent sitting at a desk, visiting friends, travelling, reading or sitting or lying down to watch television BUT NOT INCLUDING the time spent sleeping?  hours  do not know / not sure | | |

**Section 8 Complete? Yes**

**Section 9: Sleep pattern and cell phone use**

| **SL No.** | **Question** | **Code** | **Response/ Code** |
| --- | --- | --- | --- |
| 9A | About how many hours sleep do you get in every 24 hours? (including daytime naps) | \|___\|___\| hours  Do not know ………………………………………………1  Prefer not to answer…………………………………..2 |  |
| 9B | Do you have a nap during the day? | Never/Rarely…………………………………1 (*go to 9D)*  Sometimes (1-2 days per week)......2  Usually (>3 times per week).…………3  Prefer not to answer….…...…………….4 (*go to 9D)* |  |
| 9C | About how many hours do you sleep over a day-time nap? | \|___\|___\| hours  Do not know ………………………………………………1  Prefer not to answer…………………………………..2 |  |
| 9D | For approximately how many years have you been using a mobile phone at least once per week to make or receive calls? | Never use mobile phone..…………………………..1 (Go to section 10)  1 year or less.………………………………………………2  2-4 years………………...…………………………………..3  5-10 years……………...…………………………………..4  More than 10 years.…………………………………..5 |  |
| 9E | Over the last 3 months, on average how much time per week did you spend making or receiving calls on a mobile phone? | Less than 5 mins..…………………………..1  5-29 mins.………………………………………2  30-59 mins..…………………………………..3  1-3 hours....…………………………………..4  4-6 hours...…………………………………..5  More than 6 hours.……………………..6 |  |
| 9F | Over the last 3 months, on average how much time per week did you spend using your mobile phone for purposes other than making or receiving calls? | Less than 5 mins..…………………………..1  5-29 mins.………………………………………2  30-59 mins..…………………………………..3  1-3 hours....…………………………………..4  4-6 hours...…………………………………..5  More than 6 hours.……………………..6 |  |

**Section 9 Complete? Yes**

**Section 10: Food frequency questionnaire**

| Food Habit  In this section, respondents will be asked questions on whether they have eaten types of foods listed.  To complete the questionnaire please indicate i) the frequency with which respondents consumed each food (either daily or weekly or monthly) and ii) the number of servings that were eaten by the respondent each time the food was consumed. For example, If the respondent ate white bread 3 times a week, two slices each time, write down “3” in the **Weekly** column and write down “2” in the **servings each time eaten**. Within the past month, if the respondent did not eat wholemeal bread write down “0” in the **Monthly** column.   1. Every type of food has been given their typical serving size according to the ‘Malaysian Food Serving Size Album’ and also a list of weights of foods in household measurements. In order to assess the number of servings consumed, interviewers will need to show the food photos or standard food measurement tools (provided) to respondents for each meal. | | | | | | | |
| --- | --- | --- | --- | --- | --- | --- | --- |
| If the respondent took white bread 3 times a week, two slices each time, write down “3” in the **Weekly** column and write down “2” in the **servings each time eaten**. Within the past month, if the respondent did not take wholemeal bread write down “0” in the **Monthly** column. | | | | | | | |
|  | Type of food | | How frequent was each food taken  (Fill in one of the columns only) | | | Reference of meal size | Total servings  (each time eaten) |
|  | Cereals and cereal products | | Daily | Weekly | Monthly |  |  |
| **G1001** | White rice | |  |  |  | Cup |  |
| **G1002** | Flavoured Rice (Nasi Lemak/Nasi Dagang/ Nasi Briyani/ Nasi Goreng/ Nasi Kerabu / Nasi Arab, etc.) | |  |  |  | Cup |  |
| **G1003** | Noodles (Mee Kuning / Mee siput / Mee segera) | |  |  |  | Cup |  |
| **G1004** | Mihun/ kuehteow / laksa/ laksam/ lohshi fun | |  |  |  | Cup |  |
| **G1005** | Pasta | |  |  |  | Cup |  |
| **G1006** | Bread | |  |  |  | Slices |  |
| **G1007** | Pratha (roti telur /roti sardine/roti bawang/roti pisang/ murtabak) | |  |  |  | Slices |  |
|  | Fast food | | Daily | Weekly | Monthly |  |  |
| **G1008** | Burger | |  |  |  | Pieces |  |
| **G1009** | Fried Chicken | |  |  |  | Pieces |  |
| **G1010** | Pizza | |  |  |  | Slices |  |
| **G1011** | French Fries | |  |  |  | Medium size |  |
| **G1012** | Sausage/ Hotdog/ Frankfurter | |  |  |  | Slices |  |
| **G1013** | Nugget | |  |  |  | Pieces |  |
|  | Meat and meat products | | Daily | Weekly | Monthly |  |  |
| **G1101** | Chicken | |  |  |  | Pieces |  |
| **G1102** | Meat | |  |  |  | Matchbox size |  |
| **G1103** | Mutton | |  |  |  | Matchbox size |  |
| **G1104** | Internal organs (liver, spleen, lungs) | |  |  |  | Matchbox size |  |
| **G1105** | Chicken/ meat ball | |  |  |  | Pieces |  |
|  |  | |  |  |  |  |  |
|  | Meat and meat products  *Pork products, For Non-Muslims | | Daily | Weekly | Monthly |  |  |
| **G1106** | *Ham | |  |  |  | Slices |  |
| **G1107** | *Bacon | |  |  |  | Slices |  |
| **G1108** | *Luncheon Meat | |  |  |  | Slices |  |
| **G1109** | *Pork | |  |  |  | Matchbox size |  |
|  | Fish and seafood | | Daily | Weekly | Monthly |  |  |
| **G1201** | Marine fish | |  |  |  | Whole |  |
| **G1202** | Prawn | |  |  |  | Whole |  |
| **G1203** | Squid | |  |  |  | Whole |  |
| **G1204** | Crab | |  |  |  | Whole |  |
| **G1205** | Shellfish | |  |  |  | Tablespoon |  |
| **G1206** | Fish/ prawn/ squid/ crab crackers/ ‘keropok lekor’ | |  |  |  | Slices |  |
| **G1207** | Fish/ prawn/ squid/ crab ball or cake | |  |  |  | Pieces |  |
|  | Eggs | | Daily | Weekly | Monthly |  |  |
| **G1301** | Any eggs | |  |  |  | Pieces |  |
|  | Legumes and legume products | | Daily | Weekly | Monthly |  |  |
| **G1401** | Legumes (ground nuts, green bean, red bean, kacang tanah, kacang hijau, kacang merah, kacang kuda) | |  |  |  | Tablespoon |  |
| **G1402** | Taufufa | |  |  |  | Tablespoon |  |
| **G1403** | Tauhu | |  |  |  | Slices |  |
| **G1404** | Fermented soy beans/ ‘tempe’ | |  |  |  | Slices |  |
|  | Milk and milk products | | Daily | Weekly | Monthly |  |  |
| **G1501** | Fresh milk | |  |  |  | Cup |  |
| **G1502** | Commercial milk | |  |  |  | Cup |  |
| **G1503** | Yogurt | |  |  |  | Cup |  |
| **G1504** | Powdered milk | |  |  |  | Tablespoon |  |
| **G1505** | Evaporated milk | |  |  |  | Tablespoon |  |
| **G1506** | Cheese | |  |  |  | Slices |  |
|  | Vegetables | | Daily | Weekly | Monthly |  |  |
| **G1601** | Leafy green vegetables | |  |  |  | Tablespoon |  |
| **G1602** | Legumes vegetables (ladies finger, long bean, string bean) | |  |  |  | Tablespoon |  |
| **G1603** | Tubers vegetables (potatoes, sweet potatoes, yam) | |  |  |  | Tablespoon |  |
| **G1604** | Fruit vegetables (Luffa/ pumpkin/ cucumber/ baby corn brinjal, tomatoes, chillies) | |  |  |  | Tablespoon |  |
| **G1605** | Cabbages (cabbage, cauliflower, broccoli) | |  |  |  | Tablespoon |  |
| **G1606** | Local fresh salads (ulam-ulaman) | |  |  |  | Tablespoon |  |
|  | Fruits | | Daily | Weekly | Monthly |  |  |
| **G1701** | Any fruit | |  |  |  | Slices |  |
|  | Drinks | | Daily | Weekly | Monthly |  |  |
| **G1801** | Plain water | |  |  |  | Cup |  |
| **G1802** | Tea | |  |  |  | Cup |  |
| **G1803** | Coffee | |  |  |  | Cup |  |
| **G1804** | Chocolate/ malted drink | |  |  |  | Cup |  |
| **G1805** | Pre-mixed drinks (3 in 1) | |  |  |  | Cup |  |
| **G1806** | Ready to drink beverages (canned drink) | |  |  |  | Cup |  |
| **G1807** | Cordial syrup | |  |  |  | Cup |  |
| **G1808** | Fruit juice | |  |  |  | Cup |  |
| **G1809** | Carbonated drinks (includes isotonic) | |  |  |  | Cup |  |
| **G1810** | Soya milk | |  |  |  | Cup |  |
| **G1811** | Energy drinks | |  |  |  | Cup |  |
| **G1812** | Yoghurt drinks | |  |  |  | Cup |  |
|  | Alcoholic Drinks | | Daily | Weekly | Monthly |  |  |
| **G1901** | Syandi | |  |  |  | Glass |  |
| **G1902** | Bir/Lager/ale/stout | |  |  |  | Glass |  |
| **G1903** | Todi (tuakkelapa/bahar) | |  |  |  | Glass |  |
| **G1904** | Wain/cider/champagne/peri | |  |  |  | Glass |  |
| **G1905** | Wain beras/tuakberas/lihing | |  |  |  | Glass |  |
| **G1906** | Brandi/rum/wiski/vodka/gin/samsu/samcheng/montoku/langkau | |  |  |  | Glass |  |
|  | Confectionaries | | Daily | Weekly | Monthly |  |  |
| **G2001** | Local kuih | |  |  |  | Pieces |  |
| **G2002** | Sweets (include chocolate bar, jelly/ custard, lolly ice) | |  |  |  | Pieces |  |
| **G2003** | Cake | |  |  |  | Slices |  |
| **G2004** | Pastry (Pie, croissant) | |  |  |  | Pieces |  |
| **G2005** | Snacks/Crackers | |  |  |  | Pieces |  |
|  | Bread Spread | | Daily | Weekly | Monthly |  |  |
| **G2101** | Jam | |  |  |  | Teaspoon |  |
| **G2102** | Coconut jam/ Kaya | |  |  |  | Teaspoon |  |
| **G2103** | Butter | |  |  |  | Teaspoon |  |
| **G2104** | Margarine | |  |  |  | Teaspoon |  |
| **G2105** | Peanut butter | |  |  |  | Teaspoon |  |
| **G2106** | Cream cheese | |  |  |  | Teaspoon |  |
| **G2107** | Chocolate spread | |  |  |  | Teaspoon |  |
|  | Flavours | | Daily | Weekly | Monthly |  |  |
| **G2201** | Sugar (white, brown, Melaka) | |  |  |  | Teaspoon |  |
| **G2202** | Honey | |  |  |  | Teaspoon |  |
| **G2203** | Condensed milk (creamer) | |  |  |  | Tablespoon |  |
| **G2204** | Condiment (include budu, cencalok, soy sauce, chilli sauce, tomato ketchup, oyster sauce, fish sauce, petis) | |  |  |  | Teaspoon |  |
| **G2205** | Shrimp paste/ Belacan | |  |  |  | Teaspoon |  |
| **G2206** | Salad dressing | |  |  |  | Teaspoon |  |
|  | | | | | | | |
| In this section, respondents will answer the amount of sugar, oil, coconut milk and salt usually use by that household | | | | | | | |
| G2300. Sugar usage by household | | G2400. Coconut milk usage by household | | | | | |
| G2301 | How much sugar do you buy each time?  ……………….kg | G2401 | How much coconut milk do you buy each time?  ……………….kg | | | | |
| G2302 | With that amount how long does that sugar last?  ……………….days | G2402 | With that amount how long does that coconut milk last?  ……………… days | | | | |
| G2303 | Usually how many people consume that amount of sugar?  ……………….people | G2403 | Usually how many people consume that amount of coconut milk?  ……………….people | | | | |
| G2500. Salt usage by household | | G2600. Palm oil usage by household | | | | | |
| G2501 | How much salt do you buy each time?  ……………….kg | G2601 | How much palm oil do you buy each time?  ……………….kg | | | | |
| G2502 | With that amount how long does that salt last?  ……………… days | G2602 | With that amount how long does that palm oil last?  ……………… days | | | | |
| G2503 | Usually how many people consume that amount of salt?  ………………..people | G2603 | Usually how many people consume that amount of palm oil?  ………………..people | | | | |
| G2700. Sunflower oil usage by household | | G2800. Olive oil usage by household | | | | | |
| G2701 | How much Sunflower oil do you buy each time?  ……………….kg | G2801 | How much olive oil do you buy each time?  ……………….kg | | | | |
| G2702 | With that amount how long does that Sunflower oil last?  ………………. days | G2802 | With that amount how long does that olive oil last?  ……………… days | | | | |
| G2703 | Usually how many people consume that amount of Sunflower oil?  ………………..people | G2803 | Usually how many people consume that amount of olive oil?  ………………..people | | | | |
| G2900. Soya bean oil usage by household | |  |  |  |  |  |  |
| G2901 | How much Soya bean oil do you buy each time?  ……………….kg |  |  |  |  |  |  |
| G2902 | With that amount how long does that Soya bean oil last?  ……………… days |  |  |  |  |  |  |
| G2903 | Usually how many people consume that amount of Soya bean oil?  ……………….people |  |  |  |  |  |  |

**Section 10 Complete? Yes**

**Section-11: Physical Measurements**

| **SL No.** | **Question** | **Code** | **Response/ Code** |
| --- | --- | --- | --- |
| SSO6. | ER-thrombolysis time | \|___\|___\| : \|___\|___\| AM/PM  hour(s) minute(s) |  |
| SSO7. | Thrombolytic infusion | Yes 1  No 2  Not applicable 3 |  |
| SSO8. | Type of MI (Select only one) | Anterior 1  Antero-septal 2  Inferior 3  Lateral 4  Posterior 5  Right ventricle 6  NSTEMI 7  Other 8 |  |
| SSO9. | Outcome for the current event | Discharged with advice 1  Discharged on request 2  Absconded 3  Referred to other hospital 4  Death 5  Unknown 6 |  |

|  |  | First Reading | Second Reading |
| --- | --- | --- | --- |
| SS10. | Systolic blood pressure (mm of Hg) |  |  |
| SS11. | Diastolic blood pressure (mm of Hg) |  |  |
| SS12. | Heart Rate (beats/minute) |  |  |
| SS13 | Waist (taken to the nearest 1cm) |  |  |
| SS14 | Hip (taken to the nearest 1cm) |  |  |
| SS15 | Weight (taken to the nearest 0.1kg) |  |  |
| SS16 | Height (taken to the nearest 1cm) |  |  |

**Section 11 Complete? Yes**

**Section 12 – Laboratory Log Sheet**

| **Subject ID** |  |  |  |  |  |  |  |  |
| --- | --- | --- | --- | --- | --- | --- | --- | --- |
| **Column 1** | **Column 2** | **Column 3** | **Column 4** | **Column 5** | | **Column 6** | **Column 7** | **Column 8** |
| **Sample type** | **Tube type** | **Hospital ID** | **Present/Missing/Empty** | **Box Id** | | **Box position** | **Spare labels** | **Blood volume** |
|  |  |  | *Please indicate if sample tubes are present and filled (Y), or not present/empty* *(N)* | *Add box number in which tubes are placed or enter 'N' if missing* | | *Add box position in which tubes are placed or enter 'N' if missing* | *If spare labels used indicate which label (SP1 - SP4) has been used on which tube* | *Please indicate if aliquot is correct volume or more (CV), is less than CV but at least half full (>HF) or is less than CV and less than half full (<HF)* |
| EDTA tube | TE1 |  |  | n/a | | n/a |  |  |
| Serum tube | TS1 |  |  | n/a | | n/a |  |  |
| EDTA tube | TE2 |  |  | n/a | | n/a |  |  |
| Serum tube | TS2 |  |  | n/a | | n/a |  |  |
| whole blood | W01 |  |  | CAMWB |  |  |  |  |
| whole blood | W02 |  |  | CAMWB |  |  |  |  |
| whole blood | W03 |  |  | MLYWB |  |  |  |  |
| plasma | P04 |  |  | CAMPL |  |  |  |  |
| plasma | P05 |  |  | CAMPL |  |  |  |  |
| plasma | P06 |  |  | MLYPL |  |  |  |  |
| plasma | P07 |  |  | MLYPL |  |  |  |  |
| serum | S08 |  |  | CAMSE |  |  |  |  |
| serum | S09 |  |  | CAMSE |  |  |  |  |
| serum | S10 |  |  | MLYSE |  |  |  |  |
| serum | S11 |  |  | MLYSE |  |  |  |  |

**Section 12 Complete? Yes**

**
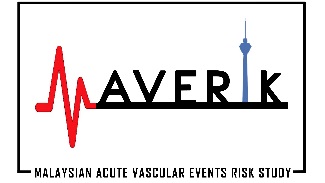
**MAV-10 – SOP of physical measurements V1 20170515

**PHYSICAL MEASUREMENTS PROTOCOL**

Members of the research team will perform physical measurements in participants in the order described using standardised protocols. Measurements will be recorded on the Maverik App (see Training Document MAV-05) after all sections of the questionnaire (up to and including the food frequency questionnaire) have been completed.

Before starting any measurements, the research team member will wash their hands and explain the procedure to the participant.

For patients (i.e. not controls): The research team member will ensure that the patient is in a stable condition before conducting any measurements. If the patient is not stable / requires medical attention, return later to make measurements. A look up table, either in a hard copy form (see Appendix 1) or as part of the APP-based questionnaire, will be available to researchers so that they are able to identify patients whose physical measurements have yet to be completed.

**1. Blood pressure and heart rate**

Two measurements of blood pressure (systolic and diastolic), plus heart rate, will be taken using Omron Blood Pressure monitor HEM 7130 (standardised across all study sites). These measurements will be recorded in the Physical Measurements Section of the MAVERK study APP.

The research team member will:

1. Introduce themselves to the participant and confirm the participant’s personal information (if this has not already been done as part of the questionnaire completion process)
2. Ensure that the participant removes any clothing with thick sleeves which may affect blood pressure measurements (rolling up thick sleeves should be avoided)
3. Ensure that the participant is in a quiet environment and is seated in a comfortable position with legs uncrossed and feet flat on the floor
4. Place the participants arm so that the antecubital fossa is level with the heart, whilst making sure that that the arm is straight but at the same time supported
5. Select an appropriate size cuff for the participant according to the circumference of the participant’s arms and place the cuff either over bare skin or light clothing
6. Make sure that the air tube is not bent and that it is on the outer side
7. Wrap the cuff around the arm taking care to attach it so that:
8. The marker (arrow under the air tube) is centred on the middle of your inner arm.
9. The bottom edge of the cuff is 2cm above the antecubital fossa ~~and~~
10. The cuff is wrapped to a tightness that roughly allows two fingers to be inserted under the cuff
11. The cuff wrapping guide lamp lights in green to indicate that the cuff is correctly wrapped tightly enough on the arm and the reading is accurate and reliable.
12. Press the [START/STOP] button
13. Wait until the measurement has finished and then record the measurement results which are displayed on the monitor in the Physical Measurements section of the MAVERIK study APP. Measurements will include systolic blood pressure, diastolic blood pressure and heart rate.
14. Repeat steps h-i above to perform a second measurement.

**2. Waist**

Two measurements of waist circumference will be measured using a SECA measuring tape. These measurements will be recorded in the Physical Measurements Section of the MAVERK study APP.

The research team member will:

1. Ensure that the participant is standing with both feet close together, has their arms by their side, has their body weight evenly distributed and is wearing thin clothing
2. Identify the mid-point between the highest point of the iliac crest and the inferior margin of the lower rib
3. Apply the tape at the mid-point and ensure that the tape is level around the waist. The tape should rest on the skin but not compress soft tissues
4. Ask the volunteer to relax, i.e. not to deliberately hold him/herself in or out, and to look straight ahead
5. Read the measurement at the end of a normal expiration. Measure to the nearest 0.1cm
6. Record the result in the Physical Measurements section of the MAVERIK study APP
7. Repeat steps b-f above to perform a second measurement.

**3. Hip**

Two measurements of hip circumference will be measured using a SECA measuring tape. These measurements will be recorded in the Physical Measurements Section of the MAVERK study APP.

The research team member will:

1. Ensure that the participant is standing with both feet close together, has their arms by their side, has their body weight evenly distributed and is wearing thin clothing
2. Apply the tape around the pelvis at the widest portion of the buttocks, with the tape parallel to the floor
3. The tape should rest on the skin but not compress soft tissues
4. Read the measurement at the end of a normal expiration. Measure to the nearest 0.1cm
5. Record the result in the Physical Measurements section of the MAVERIK study APP
6. Repeat steps b-e above to perform a second measurement.

**4. Weight**

Two measurements of weight will be measured using Electronic Column Scale (with height measurement rod) SECA 769 (standardised across all study sites) to the nearest 0.1kg. These measurements will be recorded in the Physical Measurements Section of the MAVERK study APP.

The research team member will:

1. ask participants to remove their shoes and outer garments (e.g. coats) such that the measurement is made light clothing only
2. ask participants stand on the centre of the scales, arms to the side, looking forward.
3. Then ask the participant to step off the weigh scales
4. Record the result in the Physical Measurements section of the MAVERIK study APP
5. Repeat steps b –d to perform a second measurement.

**5. Height**

Two measurements of height will be measured using Height measurement rod SECA 220 attached to the Electronic Column Scale (standardised across all study sites) to the nearest 0.1cm. These measurements will be recorded in the Physical Measurements Section of the MAVERK study APP.

The research team member will:

1. Unfold the measuring flap so that it engages in a horizontal position
2. Extend the upper end of the rod (as required) until the participant can comfortably stand under the measuring flap
3. Ask the participant to step under the measuring flap with their:

- Back to the measuring rod
- Body kept straight
- Head straight (with eyes looking ahead) parallel to the measuring flap

1. Slide down the measuring flap until it rests on the participant’s head and read off the measured result. Then ask the participant to step away from the measuring rod
2. Record the result in the Physical Measurements section of the MAVERIK study APP
3. Repeat steps c –e to perform a second measurement
